# Transcriptome Analysis of Female and Male *Xiphophorus maculatus* Jp 163 A

**DOI:** 10.1371/journal.pone.0018379

**Published:** 2011-04-05

**Authors:** Ziping Zhang, Yilei Wang, Shuhong Wang, Jingtao Liu, Wesley Warren, Makedonka Mitreva, Ronald B. Walter

**Affiliations:** 1 Department of Chemistry and Biochemistry, Molecular Biosciences Research Group, Texas State University, San Marcos, Texas, United States of America; 2 Key Laboratory of Science and Technology for Aquaculture and Food Safety of Fujian Province University, Fisheries College/Fisheries Biotechnology Institute, Jimei University, Xiamen, China; 3 Genome Sequencing Center, Washington University School of Medicine, St. Louis, Missouri, United States of America; University of Birmingham, United Kingdom

## Abstract

**Background:**

*Xiphophorus* models are important for melanoma, sex determination and differentiation, ovoviviparity and evolution. To gain a global view of the molecular mechanism(s) whereby gene expression may influence sexual dimorphism in *Xiphophorus* and to develop a database for future studies, we performed a large-scale transcriptome study.

**Methodology/Principal Findings:**

The 454-FLX massively parallel DNA sequencing platform was employed to obtain 742,771 and 721,543 reads from 2 normalized cDNA libraries generated from whole adult female and male *X. maculatus* Jp 163 A, respectively. The reads assembled into 45,538 contigs (here, a "contig" is a set of contiguous sequences), of which, 11,918 shared homology to existing protein sequences. These numbers estimate that the contigs may cover 53% of the total number of *Xiphophorus* transcriptome. Putative translations were obtained for 11,918 cDNA contigs, of which, 3,049 amino acid sequences contain Pfam domains and 11,064 contigs encode secretory proteins. A total of 3,898 contigs were associated with 2,781 InterPro (IPR) entries and 5,411 contigs with 132 KEGG (Kyoto Encyclopedia of Genes and Genomes) pathways. There were 10,446 contigs annotated with 69,778 gene ontology (GO) terms and the three corresponding organizing principles. Fifty-four potential sex differentially expressed genes have been identified from these contigs. Eight and nine of these contigs were confirmed by real-time PCR as female and male predominantly expressed genes respectively. Based on annotation results, 34 contigs were predicted to be differentially expressed in male and female and 17 of them were also confirmed by real-time PCR.

**Conclusions/Significance:**

This is the first report of an annotated overview of the transcriptome of *X. maculatus* and identification of sex differentially expressed genes. These data will be of interest to researchers using the *Xiphophorus* model. This work also provides an archive for future studies in molecular mechanisms of sexual dimorphism and evolution, and can be used in comparative studies of other fish.

## Introduction

The molecular mechanisms that control sexual dimorphism are very different in distantly related animals. However, for most of the animals, the differences between female and male result from the regulation of at least three developmental processes: 1. female and male differ in the sex determination of their somas, 2. the sexual differentiation of their germline, and 3. the level of transcriptional activity of their sex chromosomes [1]. The third level of gene activity comprises genes that encode terminal differentiation functions such as sex-specific macromolecules, structures, physiology, or behaviors.

Sex determination mechanisms among the many species of *Xiphophorus* fish are quite diverse and have been well-characterized for 12 species [2]. Both WY/YY and XX/XY mechanisms function in these species. Interestingly, at least one species (*X. maculatus*) was found possessing a multiple sex chromosome mechanism (WY, WX or XX females; XY or YY males) [3]. *X. maculatus* Jp 163 A is female homogametic (XX). *X. helleri* Sarabia which may be crossed with *X. maculatus* Jp 163 A to develop an interspecies hybrid melanoma model is male homogametic (YY) [2], [4], [5]. The diversity of sex determination mechanisms among the 26 *Xiphophorus* species suggest they may serve as excellent models to detail the molecular mechanisms that control sexual dimorphism [6], [7], [8]. As a live-bearing fish, *X. maculatus* is also an important model to study the evolution of ovoviviparity [9].

Gene expression measurements have been used to develop new biological concepts, refine disease classification, improve diagnostic and prognostic accuracy, and identify new molecular targets for drugs and clinical biomarkers [10]. Over the past decade, significant progress has been made in genome-wide gene expression profiling by the development and application of differential display [11], RNA fingerprinting [12], suppression subtraction hybridization [13], cDNA AFLP [14], cDNA microarrays [15] and others. These technologies have been used to profile gene expression patterns in gonads [16], to diagnostically distinguish different types of cancer, to validate drug target interactions, and to identify secondary drug target effects. In addition, various methods for transcript profiling have been used to analyze cellular pathways and processes after targeted perturbations of cell physiology. However, each of the above techniques has disadvantages, such as high false positive rates, and are labor intensive [17].

Use of next generation sequencing technology provides general representation of almost all the transcripts (i.e., mRNAs) expressed in specific cells or organs at particular conditions and times. Large-scale transcriptome analyses have great potential to identify the initial molecular changes accompanying gonadal differentiation [16]. Over the past three years, massively parallel DNA sequencing platforms have become available which reduce the cost of DNA sequencing by over two orders of magnitude, making global transcriptome analysis inexpensive, and widespread [18].

To gain a global view of the multiple interrelated molecular changes that relate to the sexual dimorphism in *Xiphophorus* and provide a database for future studies, we initiated a transcriptome project to obtain deep coverage of cDNAs from adult fish of different gender. To do this we employed the 454-FLX DNA sequencing platform [19]. We harvested RNA from *X. maculatus* Jp 163 A, a fish line which is highly inbred (here the 104^th^ generation) and has been used as the non-recurrent parent in many backcross hybrid melanoma models [20]. In order to increase the representation of the transcriptome, one pregnant female and two males were used for 454 sequencing. The DSN based normalization method [21] was employed to increase the probability of rare transcript representation. The data allowed us to perform subtractions and derive gene sets that appeared specifically expressed in one sex or the other. Herein, we present a comprehensive bioinformatic exploration, functional annotation, and real-time PCR validation of a subset of female- and male-enriched transcripts identified from adult stage of *X. maculatus* Jp 163 A.

## Materials and Methods

### Fish

Fish used in this project were obtained from the Xiphophorus Genetic Stock Center, Texas State University, San Marcos, TX (see, http://www.xiphophorus.txstate.edu/). The *X. maculatus* Jp 163 A fish used, were from pedigree 104(A). In order to increase the representation of transcriptome, one pregnant female (314 days old [DO] with embryos in this fish), 2 males (324 DO) were used for total RNA isolation from whole fish after removal of the stomach and intestine for 454 sequencing. Four male and 4 pregnant female 338 DO of fishes were dissected to obtain samples of testis and ovary mixed with eggs and embryos, and samples of female and male livers for RNA isolation employed in real-time PCR validation studies. All animal work has been conducted according to relevant national and international guidelines. Animal protocols were approved by the Texas State Institutional Animal Care and Use Committee (approval code: 0902_0127_02).

### RNA isolation

Total RNA was isolated using Trizol (Invitrogen, Carlsbad, CA) and was further purified using RNaeasy mini RNA isolation kit (Qiagen, Valencia, CA). The residual DNA was eliminated by performing a column DNase digestion at 37°C for 30 minutes. The integrity of RNA was determined by gel electrophoresis and its concentration was measured using a Nanodrop spectrophotometer.

### cDNA library preparation

cDNA synthesis was performed using SMART [22] cDNA amplification technique with some modifications. The first-strand cDNA was generated by utilizing the 5′Smart Oligo, 5′-AAGCAGTGGTAACAACGCATCCGACGCGGG-3′ and 3′Oligo dT SmartIIA, 5′-AAGCAGTGGTAACAACGCATCCGACTTTTTTTTTTTTTTTTTTTTTT-3′. The reaction was carried out in a 20 µl system containing 2 µg of total RNA. 20 µl of the first-strand cDNA synthesis product was used as template for the 1^st^ run of long-distance-PCR (LD-PCR) with optimized cycle number to amplify cDNA for normalization. SmartIIA 5′-AAGCAGTGGTAACAACGCATCCGAC-3′ was used as primer in LD-PCR. The volume of each reaction was 100 µl. The products of the 1^st^ run of LD-PCR were purified using QIAquick PCR Purification Kit. Equal amount of the purified LD-PCR products from each RNA sample was mixed for normalization. TRIMMER cDNA Normalization Kit (Innovative Biotechnology Company, Moscow, Russia) that uses duplex-specific nuclease (DSN) treatment [21] was used for normalization reaction. The normalized cDNA was used as template for the 2^nd^ run of LD-PCR. The SMART II was used as the primer for LD-PCR, and products were purified using QIAquick PCR Purification Kit.

### 454 sequencing, assembly

Sequence reads were generated from gender specific cDNA libraries on the 454 Life Sciences FLX instrument. All reads were subjected to *de novo* assembly with the version 2.0.1 of Newbler (Roche 454 Life Sciences). Assembly contigs are reported as large contigs (>500 base pair) and all contigs, singletons separately. The all contigs file was used for all subsequent analysis. The singletons file was used for analysis of function only.

### Analysis of function

All the contigs were submitted for homology and annotation searches, and Gene Ontology (GO) annotation using an online version of the BLAST2GO program (www.Blast2GO.de; Oct. 2009; see [23]. In these searches the BLASTX cut off value was set to 10^−3^. For the GO mapping process, previously validated settings were used: E-value 10^−5^, annotation Cut Off "45" and GO Weight "20". This program also utilizes EST InterProScan (http://www.ebi.ac.uk/InterProScan/) for amino acid sequence analyses. The annotation step of the program retrieves keywords in the BLASTX descriptions and converts them into Gene ontology (GO) related terms associated with homologies identified with NCBI's QBLAST and returns a list of GO annotations represented as hierarchical categories of increasing specificity. Placement into metabolic pathways was accomplished with tools supplied by the Kyoto Encyclopedia of Genes and Genomes (KEGG) (Oct., 2009), located at the KEGG Automatic Annotation Server (KAAS), http://www.genome.jp/kegg/kaas/. The data presented herein represent level 2 analyses, illustrating general functional categories. The EST reads and cDNAs were processed using the bi-directional best-hit method (forward and reverse reads) to assign orthology. KAAS provides functional annotation of putative genes by BLAST comparison against the KEGG GENES database. The output includes KO (KEGG Orthology) assignments and automatically generated KEGG pathways that are populated with the KO assignments. The program clusters the results using their semantic similarity [24], accuracy weight and the path from the root node of the ontology to the most detailed annotation. Annotation results were then used to retrieve keywords to identify genes related to sex differentiation. The 248 core eukaryotic genes [25] were used as a reference to estimate the coverage of cDNAs to the transcriptome of *X. maculatus* Jp 163 A.

### Identification of male and female differentially expressed genes

A Poisson-based enrichment test, considering both the total sampling sizes and random variations [26], was utilized to calculate the likelihood of gender specific enrichment for each cDNA contig. The test identifies differential expression from cDNA profiles by calculating the probability of counts from one sex when the counts from the other sex are known, with the assumption that the counts originated from the same distribution. A P-value cutoff of 0.001 was chosen to define the putative gender-enriched genes from the *Xiphophrus* datasets. A set of keywords, composed by sex, egg, ovary, sperm, testis, female, and male were used to predict sex differentially expressed genes based on annotation results.

### Quantitative Real-time PCR (qRT-PCR) analyses

For quantitative real-time PCR (qRT-PCR), methods described in our previous article [27], [28] were employed in this study. Primers (listed in Table S1) based on the *Xiphophorus* target genes were designed using an online real time PCR primer design tool (http://www.genscript.com/cgi-bin/tools/primer_genscript.cgi). To perform qRT-PCR, 2 µg of DNA free *Xiphophorus* RNA from different tissues of 4 pregnant female and 4 male 338 DO of fishes was reverse-transcribed using MMLV reverse transcriptase (Invitrogen). The fast SYBR green master mix (Applied Biosystems) was used for qRT-PCR. PCR products were quantified using an Applied Biosystems Prism 7500-fast real-time PCR system. The PCR reactions were initiated with denaturation at 95°C for 5 min; followed by 40 amplification cycles at 95°C for 15 s and 60°C for 30 s. Dissociation protocols were used to measure melting curves and to control non-specific signals from the primers. Following amplification, to further confirm the presence of a single amplification product, PCR products were subjected to separation on a 2% agarose gel stained with ethidium bromide. The comparative threshold cycle (CT) method was used to calculate the relative concentrations. This method involves obtaining CT values for the target gene and normalizing to the 18S rRNA from the same sample; followed by comparing the relative expression levels among samples from female and male. The quantification of gene expression was analyzed and reported as a relative quantity (RQ) to the control value, and all experiments were repeated with three or four biological replicates. The statistical package GraphPad Prism (GraphPad Software, Inc.) was used to analyze the data from all experiments. The averages of the relative quantities for the biological replications (3–4) were used in a 2-tailed student t-test at a 95% confidence level (p<0.05) to determine the significance of the difference between gene expression values in female tissues versus male tissues.

## Results

### Sequencing and assembly of X. maculatus Jp *163 A cDNAs*

In order to obtain as many as possible transcripts of *X. maculatus*, two normalized cDNA libraries were constructed from RNA isolated from whole animal female and male fishes. Four 454-FLX sequencing runs (two runs for female and two for male) were performed on the female and male normalized cDNA pools generating 777,070 and 746,074 reads, respectively (All the sequence reads are published in GenBank with accession number of SRP004487). About 95.6% and 96.7% of reads passed the quality control indices and assembled into 45,538 cDNA contigs totaling to 2.34 Mbp. The longest contig is 3977 bp. Contigs with the length of 250 bp make up the majority of assembled contigs. The size distribution of assembled contigs is presented in Figure S1.

### Functional classifications of predicted proteins

Using Blast2go, this project was able to assign gene ontology classes with BLAST matches to known proteins. The 45,538 contigs were BLASTed against the NCBI nr database and the E-value, similarity and top-hit species distributions of the hits are shown in Figure S2. There are 45,513 contigs out of the 45,538 contigs that have translations. The top-hit species in the annotated distribution was *Tetraodon nigroviridis* followed by *Danio rerio*. Of the total, 11,918 contigs (26%) were annotated with protein-coding genes from other species. Given estimates of the number of protein-coding genes in vertebrates (e.g. fish and human genome) of about 20,000–33,609 [29], [30], [31], suggests that the *X. maculatus* annotated contigs represent ≈35–60% of the total number of protein-coding *Xiphophorus* genes. Using the 248 core eukaryotic genes [25] as a reference, contigs of *X. maculatus* obtained in this project cover 53% of the *X. maculatus* transcriptome (category 1∶24 out of 66, category 2∶32 out of 56, category 3∶35 out of 61, category 4∶41 out of 65, total CEG248: 132 out of 248. 92 of those only had a single hit).

Gene ontology (GO) assignments and enzyme classifications (EC) were used to classify the functions of the predicted proteins from the *X. maculatus* contig set. In total, 10,446 contigs were annotated with 69,778 GO terms. At least one Biological Process (P) is proposed for 8,541 contigs, a Cellular Component (C) for 8,630 contigs, and 9,111 contigs were annotated with at least one Molecular Function (F). There were 6,757 contigs with annotations for all three GO categories (P, C and F), 1,055 contigs had annotations for both P and F, 705 contigs for both F and C, 564 contigs for P and C, 167 contigs for P only, 604 contigs for C only, and 594 contigs for F only. GO classifications of the contigs are shown in Figure S3. There were 2,543 contigs annotated with 2,543 Enzyme Codes. The InterPro database was also used to classify likely functions of predicted proteins. Results indicate 3,898 contigs were annotated with 2781 InterPro (IPR) items, while 5,411 contigs were annotated within 132 KEGG pathways. SignalP software predicts the presence of putative signal peptides within 11,064 contigs indicating that they may represent genes encoding secretory proteins. Also, 6,222 contigs were assigned transmembrane domains using the TMHMM (a program for prediction **t**rans**m**embrane **h**elices based on a hidden **M**arkov **m**odel).

The lengths of the longest and shortest annotated sequences were 3,055 and 93 bp respectively (Figure S4). Among unannotated sequences, 10,416 of them (31%) were equal or longer than 500 bp. Based on the annotation results, 18 genes were predicted to be involved in biological processes related to the epidermal growth factor receptor, 12 genes related to pigmentation, 385 genes related to cell proliferation, 73 genes related to DNA repair, and 54 potential sex differentially expressed genes were identified from these contigs.

### Sex-enriched gene expression and confirmation by real-time PCR

Poisson-based enrichment testing identified 2,250 male-enriched and 2,304 female-enriched contigs (enriched with a significance level of 0.001, Table 1) with various degrees of difference. Those contigs that showed robust changes in transcriptional expression (exhibited at least 100 folds enrichment of sequences in one sex over the other) were selected for further qRT-PCR validation. Among these contigs, 22 were female-predominant transcripts and 23 were male- predominant transcripts. Also, 8 female-predominant transcripts and 9 male-predominant transcripts, were confirmed as sex differentially expressed genes using independent real-time PCR analysis. Contigs predicted to be differentially expressed in male and female fish based on the annotation results, were confirmed by real-time PCR (Successfully validated contigs in testis and ovary mixed with eggs and embryos, female liver and male liver were listed in Table S2. Others were listed in Table S3). The complete list of contigs (together with their full sequences) was listed in the Supporting Text S1.

**Table 1:** Identification of male and female differential expressed genes by counting reads using Poisson-based enrichment testing.*

Total number of reads	1,464,314
number of male reads	721,543
number of female reads	742,771
number of Contigs from assembly	45,538
number of male reads in contigs	712,570
number of female reads in contigs	734,281
number of Mixed contigs	26,001
number of male only contigs	9,484
number of female only contigs	10,053
***Among the mixed contigs,***	
**Enriched with significance 0.001**	
Male enriched contigs	2,250
Female enriched contigs	2,304

*The test identifies sexual differential expression from cDNA profiles by calculating the probability of reads counts from one sex when the counts from the other sex are known, with the assumption that the counts originated from the same distribution. A P-value cutoff of 0.001 was chosen to define the putative gender-enriched genes from the *Xiphophorus maculatus* Jp 163 A datasets.

Finally, the focus of the article is the genes differentially expressed between the sexes. Since singletons do not have enough numbers for statistical analysis, we did not present those data in the main body of this manuscript. However, as the transcriptome of the species is virtually unknown, ontological analysis of these 17.5 thousand ESTs singletons was also processed in order to provide information for researchers using *Xiphophorus* models. Among these singletons, 31,374 of them were annotated with protein-coding genes (Table S4).

## Discussion

The central aim of this project was to obtain deep transcript coverage of *X. maculatus* Jp 163 A. Therefore we utilized adult male and female whole animals as the RNA source for these studies. The DSN based normalization method [21] was employed to increase the probability of rare transcript representation. While this normalization may be expected to reduce the representation of differentially expressed genes in male and female, the deep sequence analyses provided by the 454 sequencing platform were able to produce data exhibiting some gender specific gene transcripts. These sex-enriched transcripts may be rare but can be identified by comparing read counts from male and female normalized cDNAs.

About 77% of assembled contigs could not be annotated using the public databases [23]. These may correspond to 3′ or 5′ untranslated regions, non-coding RNAs, or short sequences not containing known protein domains. Considering that, in general, the longer the sequence the higher the chance of annotation (Figure S4) and number of GO terms recovered, we expect that a large number of these unannotated contigs (i.e., those longer than 500 bp) may correspond to novel or undescribed genes. Such a high percentage of novel genes may justify a deep sequencing coverage since it seems likely that “novel” ESTs would be found in more rarely expressed genes.

We performed read count analyses to identify differentially expressed ESTs based on sex. The number of sex differentially expressed genes identified by Poisson-based enrichment [26] was over 2,250 in each gender. Read count data suggested several genes that should show a robust (i.e., significance level of 0.001 and read counts larger than 100 in each gender) differential expression pattern based on sex. We chose those genes for validation using real-time PCR test and results showed that 7 of 21 tested genes in female and 9 of 24 tested genes in male corresponded with Poisson-based enrichment results. Some genes which failed to be confirmed by real-time PCR could be the result of potential male embryos present in the pregnant female, another possibility is that the sources of RNA are different: RNA for 454 deep sequencing was isolated from whole fish while RNA for qRT-PCR was isolated from dissected organs. Different sex differentially expressed genes expression level in different organs are different. Some genes are differentially expressed in other organ(s), not the gonad or liver. The results support our hypothesis that even with the process of cDNA normalization, abundant transcripts are represented more frequently in final sequenced cDNAs than rare ones. It also implies that there is potential for discovery of new genes in this organism and possibly of new gene networks and metabolic pathways using the assembled data. This is the first large EST dataset available for analyzing the *Xiphophorus* trancripts.

One of the most important aspects in mining EST data is to associate individual sequences and related expression information with biological function. In total, 10,446 contigs were annotated with 69,778 GO terms that involved binding, catalytic activity, transport, metabolism, response to stimuli, signal transduction, nucleic acid processes, and cellular biogenesis. Most of these GO ontology results were further confirmed with InterPro, Enzyme Code, KEGG, SignalP, and TMHMM mapping [23]. These annotation results allowed us to further categorize genes involved in biological processes related to sexual dimorphism.

Several vitellogenin genes were predicted as differentially expressed in female and male fish and were then further confirmed by real-time PCR. The vitellogenin a (contig00138) and vitellogenin (contig03795, contig44670) transcripts were predominantly expressed in ovary/egg. However, for vitellogenin b (contig01936), no significant difference of expression levels was observed in testis and ovaries. All of these vitellogenins were expressed in female liver with a level significantly higher than in male liver. As the precursor of a major egg yolk protein, vitellogenin is normally observed to be synthesized in the liver [32], then transported to the ovaries where it is sequestered to serve as an energy reserve for the developing embryo. A number of various vitellogenin genes have been identified in different species of fishes [32], [33], [34], [35], [36], [37], [38] and all of these reports show the expression of vitellogenin to be liver specific (or predominant). Since these reports all stem from vitellogenin genes in oviparous fishes, this may hallmark a difference in tactics by viviparous fishes such as *Xiphophorus*. Recently published research demonstrates that progressively lost ancestral vitellogenin genes relate to the origin of viviparity and placentation in mammals [39]. The research inspires us to presume that the difference in the expression patterns of vitellogenin genes in *Xiphophorus* from egg-laying fishes, may relate to the evolution of ovoviviparity. Further investigation will be performed.

Several “sperm” related genes, except sperm autoantigenic protein 17 (Sap17, contig30647) which encodes a protein present at the cell surface and is involved in fertilization by binding to the zona pellucida of the oocyte [40], were predominantly expressed in the testis. Others, such as spermatogenesis associated 13 (contig22779), spermatogenesis associated 5-like 1 (contig00883), sperm acrosomal membrane protein 14 (contig04624) are all expressed in an equatorial distribution post-acrosomal reaction and involved in spermatozoa–egg interaction [41]. Sperm adhesion molecule 1 (contig03679) which represents a new category of spermatid-expressed genes is testis-specific [42]. Nuclear autoantigenic sperm protein (contig06905), a histone binding protein that binds H1 linker histones *in vivo* and is proposed to transport histones to the nucleus of dividing cells [43], is predominately expressed in the ovary/egg/embryo rather than the testis. This contradiction in expression may have occurred in our data due to the potential mixing of developing male and female embryos at different developmental stages when RNA was isolated from the gravid *X. maculatus* female.The finding of *Sry* (Sex-determining region on the Y chromosome) [44] in mammals is a landmark discovery in biology of reproduction since it has begun to unlock the conundrum of sex determination in mammals. However, the cascade of putative molecular events initiated by *Sry* that are related to sex differentiation remain unknown [16]. It is generally accepted and supported that *Sry* evolved from the *sox 3* gene [45], [46] not earlier than 200 million years ago in the eutherian lineage [47]. There is no *Sry* in fish and it is clearly absent in *Platypus*, *Chicken*, *Zebrafinch*, *Anolis*, *Xenopus* and all the fish genomes [7], [8]. Two *Sry-*like EST sequences [*Sry* (sex determining region y)-box 5, *Sry* (sex determining region y)-box 4] were identified in this *Xiphophorus* EST data (Table S2). Real-time PCR results demonstrated that their expression level in ovary/embryo is higher than in testis. Further definition of the functions of these *Sry-*like EST sequences in sex determination in *Xiphophorus* may help illuminate the origination of sex determination genes in vertebrates.

### Conclusion

This is the first report of an annotated overview of the *X. maculatus* transcriptome and identification of sex differentially expressed genes. These data will be of considerable interest to researchers using the *Xiphophorus* model system. So far there has been little genomic information available for this fish. This work also provides an archive for future studies in molecular mechanism of sexual dimorphism and evolution, and can be also used for future *de novo* sequencing of other fish. The work also shows the enormous power of the new sequencing technology.

## Supporting Information

Figure S1 **Size distribution of assembled contigs (≥50 bp).** Sizes of contigs were counted and the graph was generated by the Blast2GO. The longest contig is 3977 bp. Contigs with the length of 250 bp occupy the majority of assembled contigs.
(PDF)

Figure S2 **Distribution of E-values (a), percent similarity (b), and top-hit species (c) from the top hit in the non-redundant protein database.**
(PDF)

Figure S3 **Pie charts of 3^rd^ level gene ontology (GO) terms from** ***Xiphophorus maculatus*** **Jp 163 A** **unicontigs.** Overall, 11,918 unique sequences were annotated using the Blast2GO software and included in the graphs. Each of the three GO categories is presented including A: Molecular Function, B: Biological Process, C: Cellular Component. Percentages are in reference to total GO-slim annotations for each category of GO-slim. Total number of annotated contigs in each category is also shown. Not all unique sequences could be annotated and some received multiple annotations.
(PDF)

Figure S4 **Percentage of contigs with length annotated.** The percentage of annotated contigs increases with the length of contigs in a fashion of linear.
(PDF)

Table S1 (DOC)

Table S2 (DOC)

Table S3 (DOC)

Table S4 (XLS)

Text S1 (DOC)
