# Supplementary figures and images for: Transcriptome Analysis of Female and Male Xiphophorus maculatus Jp 163 A
Source: PLoS One. 2011 Apr 5;6(4):e18379. doi: 10.1371/journal.pone.0018379 (PMC3071723; doi:10.1371/journal.pone.0018379)

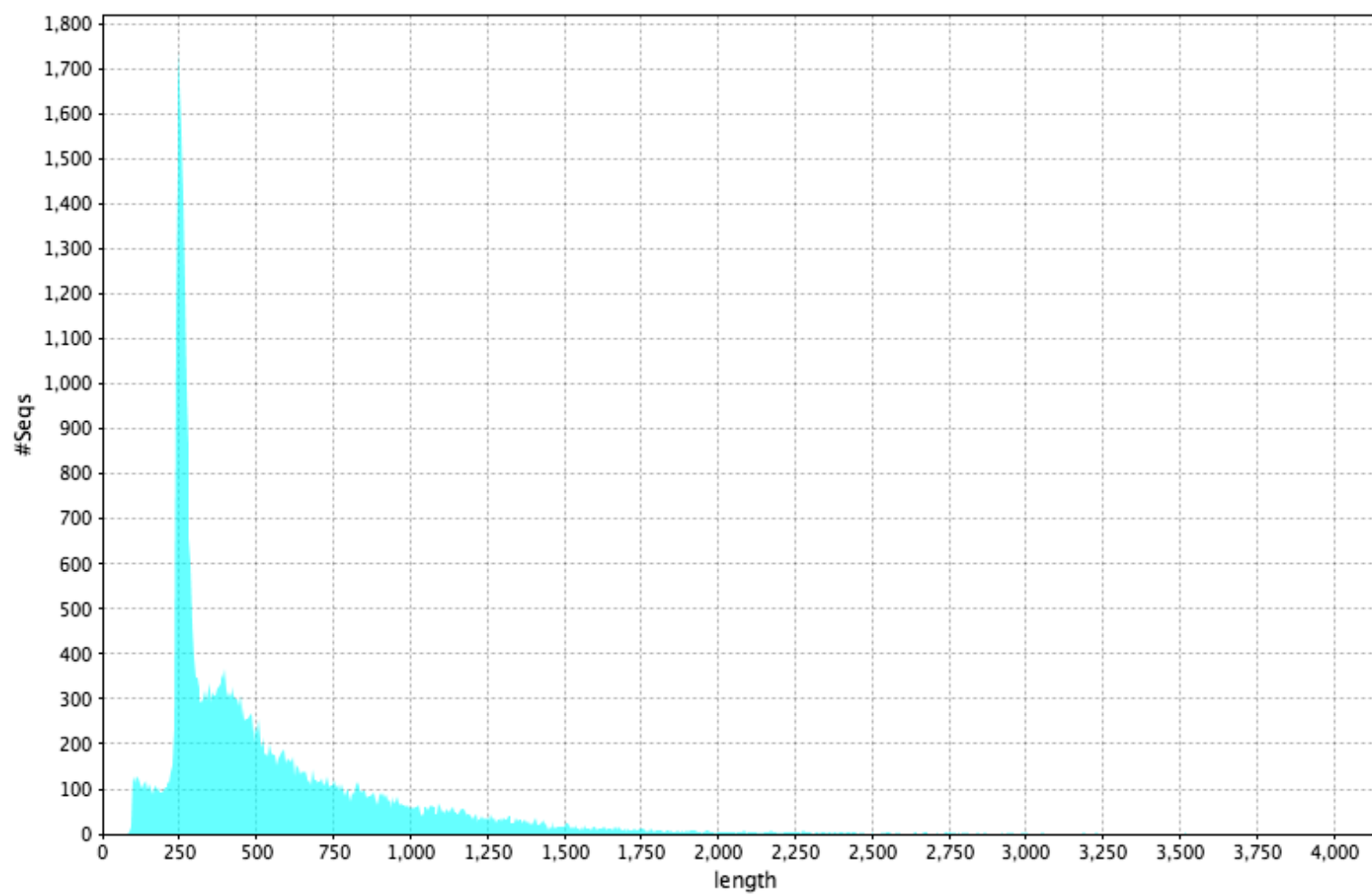

Figure S1.

Supplement: Figure S1 — Size distribution of assembled contigs (≥50 bp). Sizes of contigs were counted and the graph was generated by the Blast2GO. The longest contig is 3977 bp. Contigs with the length of 250 bp occupy the majority of assembled contigs. (PDF) [file pone.0018379.s001.pdf]

A

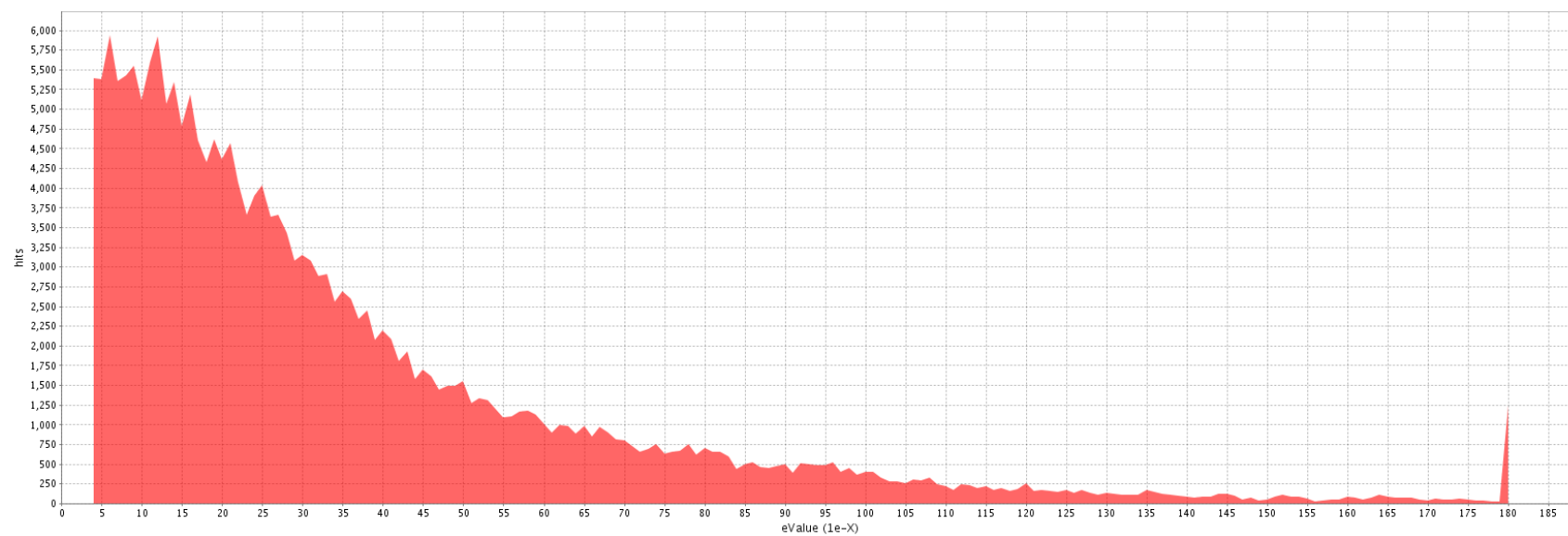

B

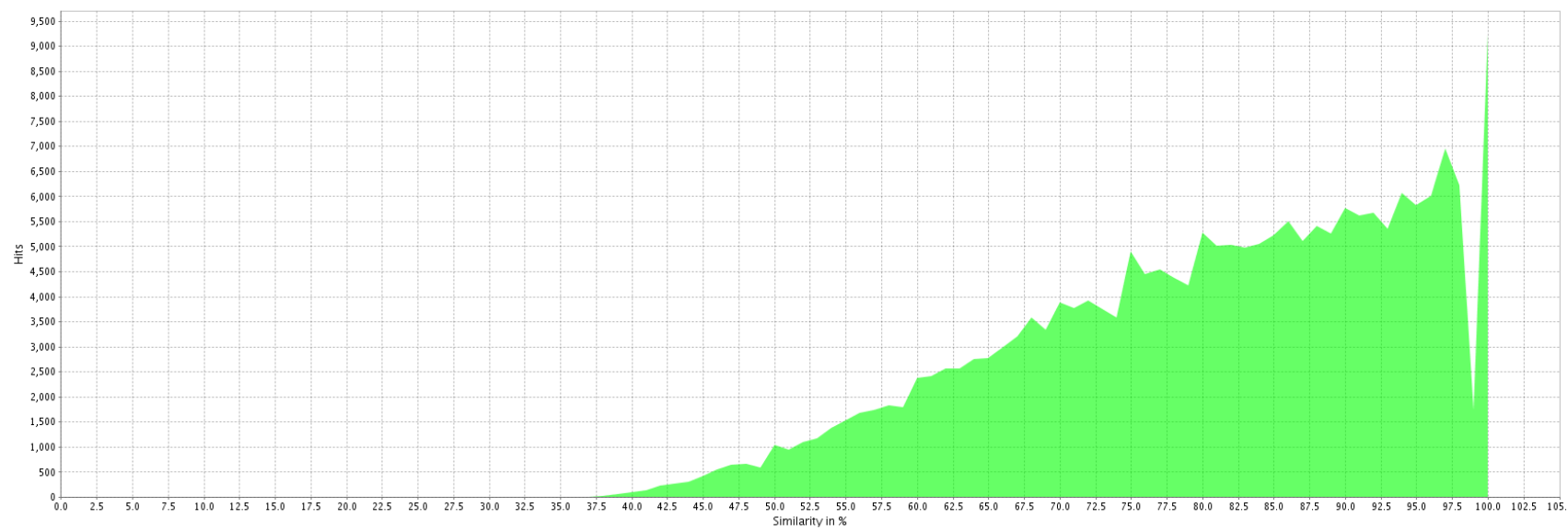

C

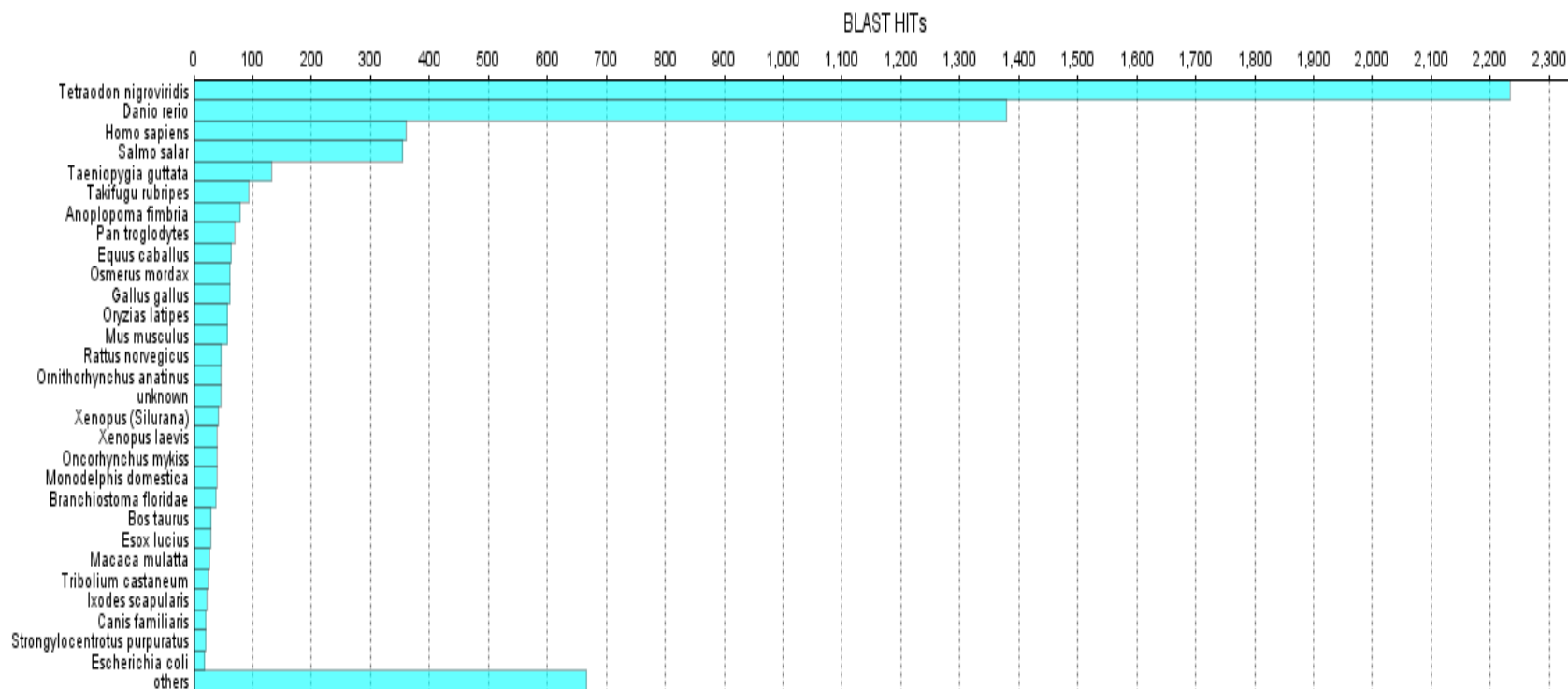

Figure S2

Supplement: Figure S2 — Distribution of E-values (a), percent similarity (b), and top-hit species (c) from the top hit in the non-redundant protein database. (PDF) [file pone.0018379.s002.pdf]

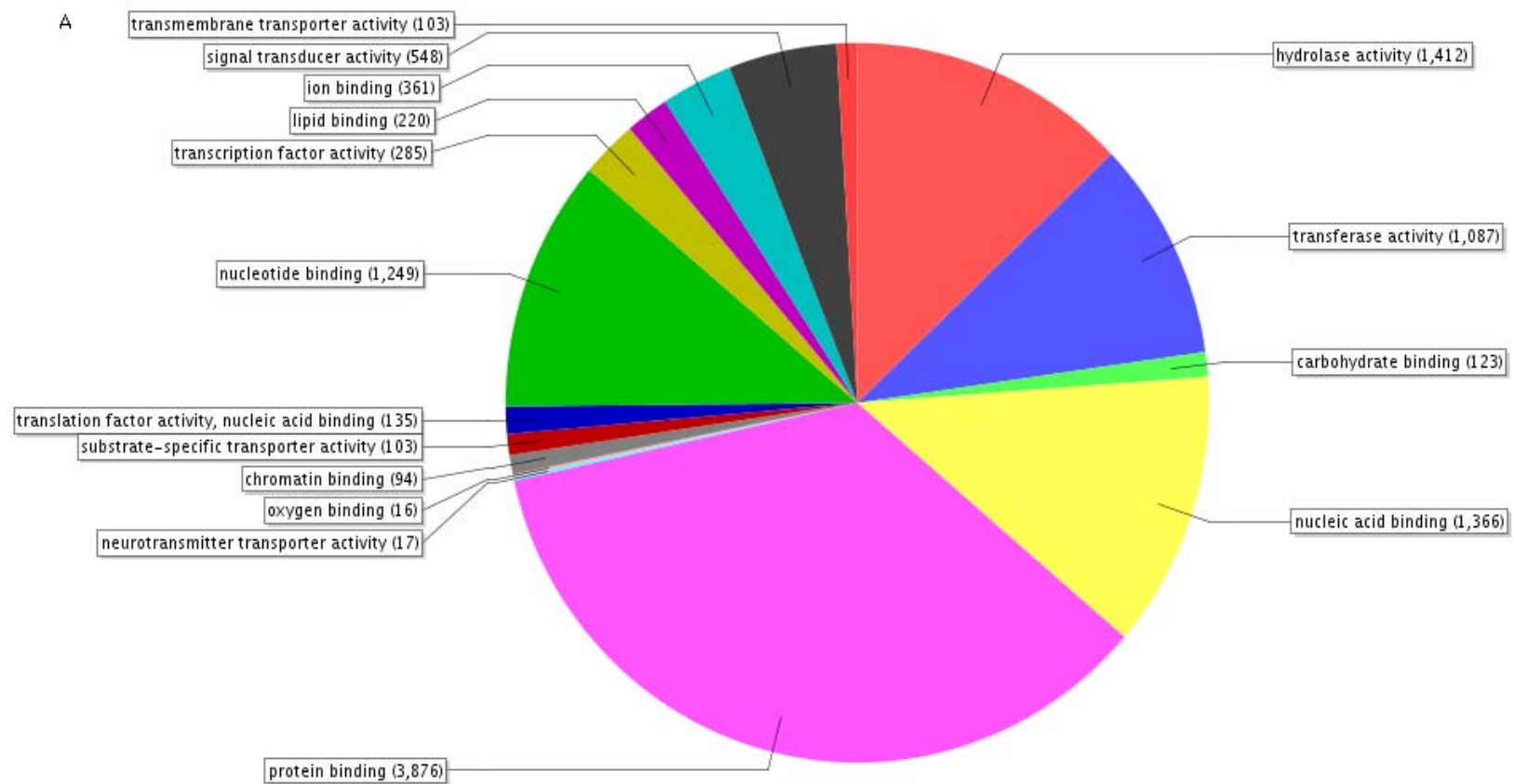

B

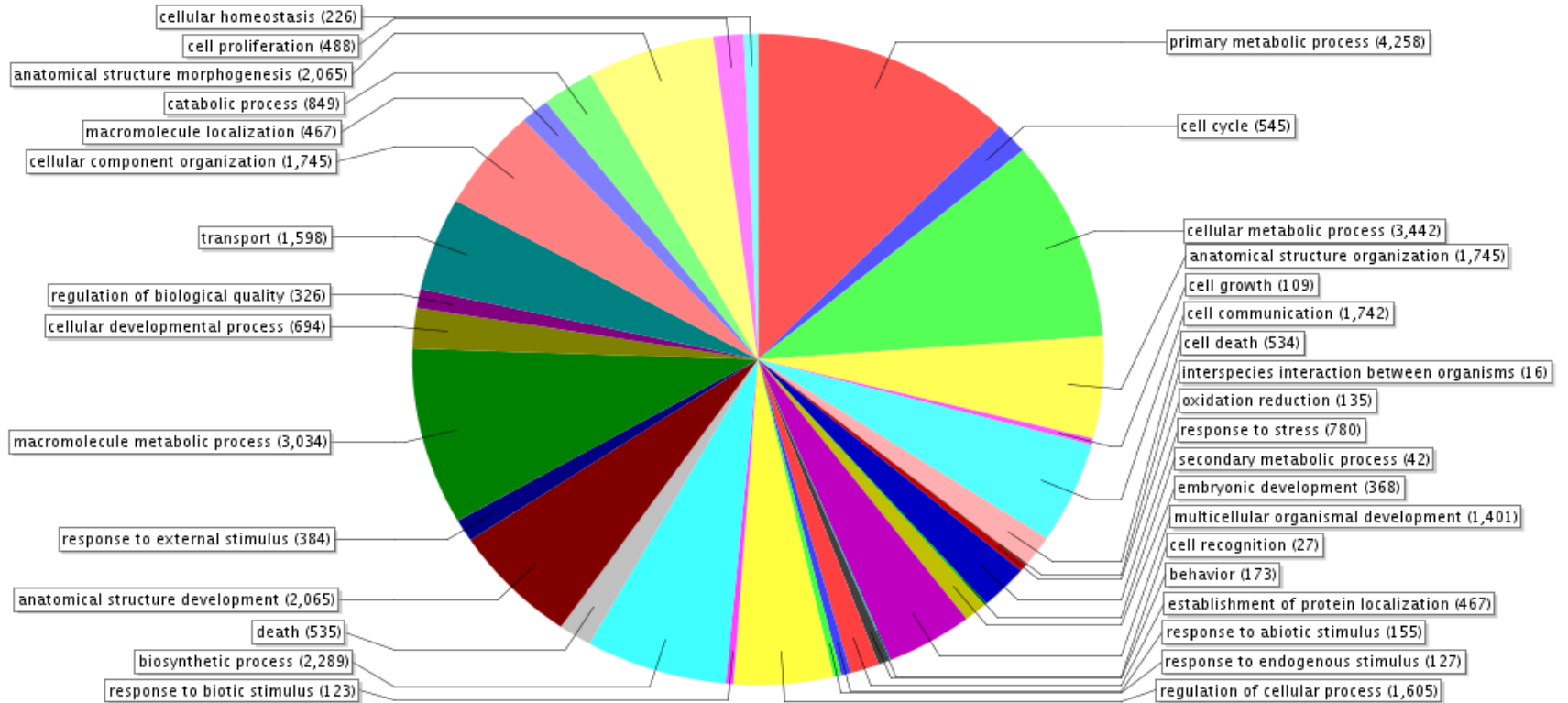

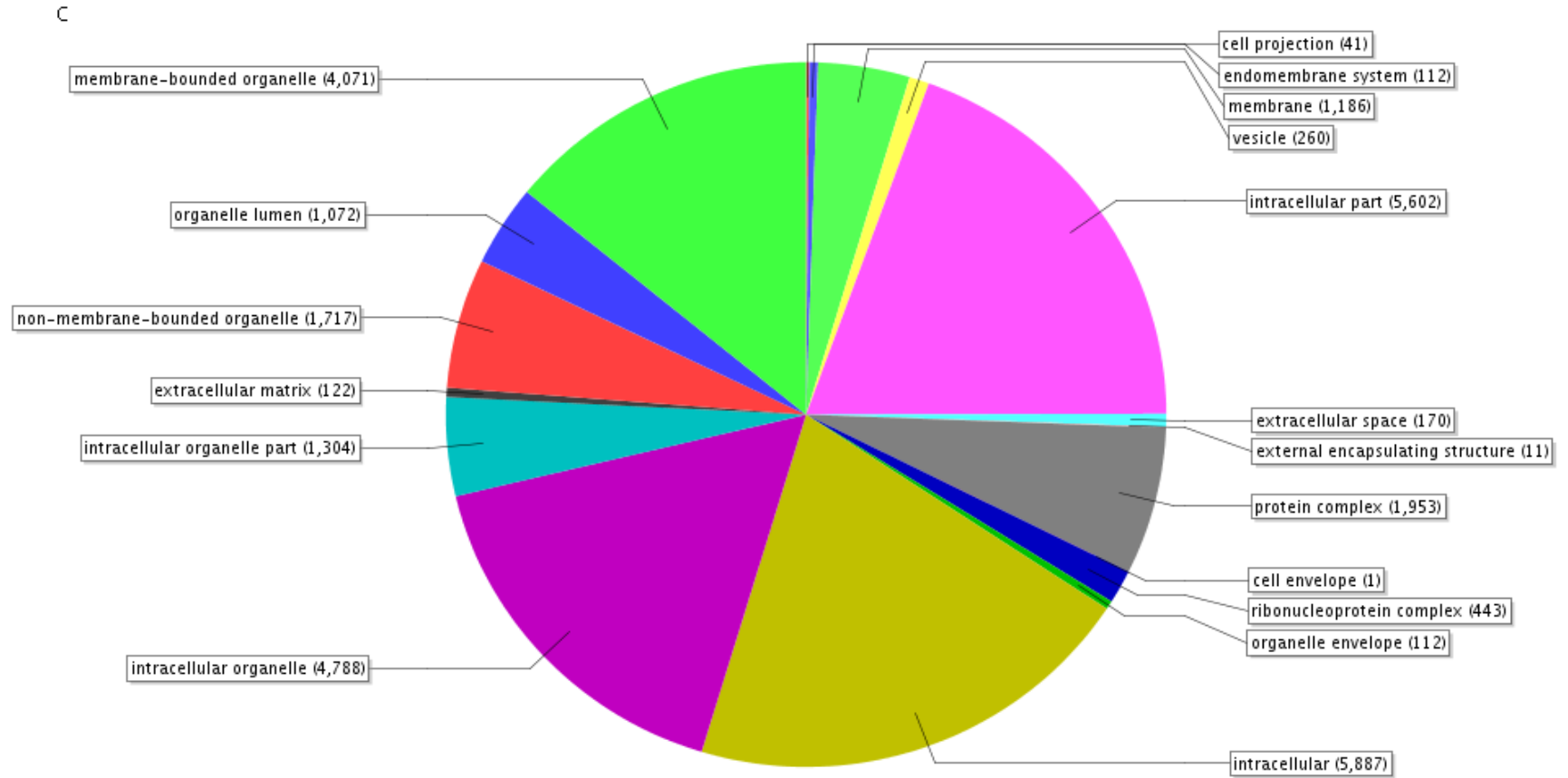

**Figure S3**

Supplement: Figure S3 — Pie charts of 3rd level gene ontology (GO) terms from Xiphophorus maculatus Jp 163 A unicontigs. Overall, 11,918 unique sequences were annotated using the Blast2GO software and included in the graphs. Each of the three GO categories is presented including A: Molecular Function, B: Biological Process, C: Cellular Component. Percentages are in reference to total GO-slim annotations for each category of GO-slim. Total number of annotated contigs in each category is also shown. Not all unique sequences could be annotated and some received multiple annotations. (PDF) [file pone.0018379.s003.pdf]

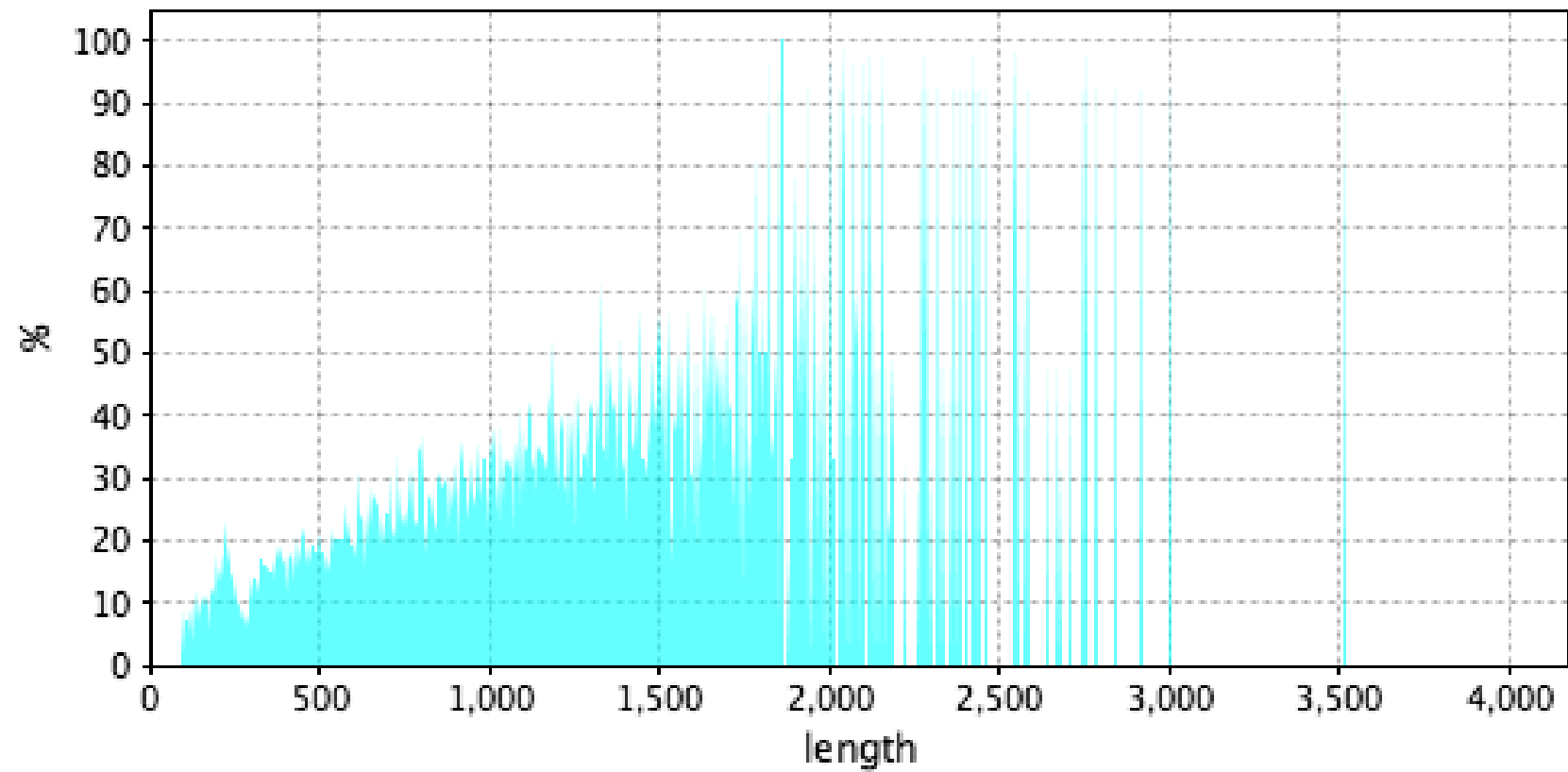

Figure S4

Supplement: Figure S4 — Percentage of contigs with length annotated. The percentage of annotated contigs increases with the length of contigs in a fashion of linear. (PDF) [file pone.0018379.s004.pdf]
