# Supplementary material for: Transcriptome Analysis of Female and Male Xiphophorus maculatus Jp 163 A
Source: PLoS One. 2011 Apr 5;6(4):e18379. doi: 10.1371/journal.pone.0018379 (PMC3071723; doi:10.1371/journal.pone.0018379)
Supplement: Table S1 — (DOC) [file pone.0018379.s005.doc]

**Table S1 Primer for real-time PCR**

| ID of Primer | Sequence of primer |
| --- | --- |
| contig00138F: | 5'-TAG ACA TGG GAA GGC GCA CA-3' |
| contig00138R: | 5'-CCT CAG AGC GTC CTG GGA AA-3' |
| contig00883F: | 5'-CAG CTG GAC TGT CAG GAG GTT TG-3' |
| contig00883R: | 5'-GGA GGA GGG CGC TGT CGA TAC-3' |
| contig01721F: | 5'-TCC TCC GAT TCT CCG TCA CCA-3' |
| contig01721R: | 5'-CCC GCC TTT GCT TGG TTT CC-3' |
| contig01827F: | 5'-TTG CTC GCT CCA CAG CCT CA-3' |
| contig01827R: | 5'-CAG CTG CAG GAC CCG GTG TT-3' |
| contig02181F: | 5'-TGT GGT GGT CGG TGC TGG AG-3' |
| contig02181R: | 5'-GGG GTG ATC GCA GTG TGA CG-3' |
| contig02487F: | 5'-TGG GCA TGA CGG ACG TGT TC-3' |
| contig02487R: | 5'-GCC TTG TGG GCC ACC GTA GA-3' |
| contig02933F: | 5'-CGG AGC CCC TAC AGC ACA GC-3' |
| contig02933R: | 5'-AGG GGT GGA AGC CCT GTA CG-3' |
| contig03679F: | 5'-GGA ATC CTG GCG CCC TCT GT-3' |
| contig03679R: | 5'-TTT GCC GAT GCC AAT GCT GA-3' |
| contig03795F: | 5'-TCG GCC GAG CTG ACC AAA AT-3' |
| contig03795R: | 5'-CAG GGC TGC CGT CAA CTT GG-3' |
| contig04624F: | 5'-GCT GGC GTT GGT CCT TGG TG-3' |
| contig04624R: | 5'-AAC CAT CGC TGC GCC AGT TC-3' |
| contig05309F: | 5'-GCT GCA ATC AAG GCC ACC ATC-3' |
| contig05309R: | 5'-GAC AGA GAA AGC CAG GCG TTT GA-3' |
| contig06275F: | 5'-GGG GGC ACA GAG CAG CAG TT-3' |
| contig06275R: | 5'-CGT CCA CTG GGC AAG ATC ACA-3' |
| contig06905F: | 5'-CGG CTG GAG ATG ACG GTG CT-3' |
| contig06905R: | 5'-GGC CGT CCT CTG GCT CTC CT-3' |
| contig07134F: | 5'-AAA GCG CGC AGA TCC AGA CG-3' |
| contig07134R: | 5'-ACG GCC GAC ACC ACC ATC AT-3' |
| contig07643F: | 5'-GCA CAG GAT CTT GTC GCC ATC A-3' |
| contig07643R: | 5'-GTG GCA TCA GCC GTG CTT CA-3' |
| contig07774F: | 5'-TGC CCC CAG CAA TCT CAT CC-3' |
| contig07774R: | 5'-CGC CGC CTC ACA TTC TCA CA-3' |
| contig10040F: | 5'-TCC GGC GAT GGC TAT GCA AC-3' |
| contig10040R: | 5'-CCC TCT GCA GGG CTG GTT GT-3' |
| contig14446F: | 5'-GCA GGG ACG GGG TTT GCA GT-3' |
| contig14446R: | 5'-CTG GGA CGT GAA CGG CGA GT-3' |
| contig15766F: | 5'-GCT CGC CGA GGT GTC TCT GG-3' |
| contig15766R: | 5'-TGC CGT GCC ACG TTC CTG TA-3' |
| contig22779F: | 5'-AAG GCG CTG CGA GTG ATG CT-3' |
| contig22779R: | 5'-ATT CCC ACC AGC GTG CCT CA-3' |
| contig26089F: | 5'-CCG CCT TCT CCC CTC TCC AC-3' |
| contig26089R: | 5'-GAC GAG GGG GCT CAC ACC AA-3' |
| contig30647F: | 5'-CGT GGG CCT CCT CTG TTG CT-3' |
| contig30647R: | 5'-GGA CCA GCC CGA CAA CAT CC-3' |
| contig30745F: | 5'-GCT GCA GGA GCT GTC AGA GGT G-3' |
| contig30745R: | 5'-GCA TGC CAA GGC CTG ACT GG-3' |
| contig32045F: | 5'-GCA GAA GCC GAG AGC GTC AA-3' |
| contig32045R: | 5'-AGG CTT TGG GGA GGG GGA TA-3' |
| contig33067F: | 5'-GAT GGC TGC TGC CCT GTT CA-3' |
| contig33067R: | 5'-CAC TGC CAC CGT CAC AGC AA-3' |
| contig40031F: | 5'-CTG CGC ATC GTC CCA ACA TC-3' |
| contig40031R: | 5'-TGG GTG GAC GGA AGC CCT TT-3' |
| contig44670F: | 5'-TGG CCA CCA AAC CCA TGG AA-3' |
| contig44670R: | 5'-GCA CTG CCG CTG CAT CTC TG-3' |
| contig45247F: | 5'-TGC TTT AAG CCG CAG CCT CTG-3' |
| contig45247R: | 5'-TGT GGA GCG ATG GAA CCC CTA-3' |
| Xiph 18s-F: | 5'-CGG AAA GGA TTG ACA GAT TGA-3' |
| Xiph 18s-R: | 5'-CTC AAT CTC GTG TGG CTG AA-3' |
