# Supplementary material for: Transcriptome Analysis of Female and Male Xiphophorus maculatus Jp 163 A
Source: PLoS One. 2011 Apr 5;6(4):e18379. doi: 10.1371/journal.pone.0018379 (PMC3071723; doi:10.1371/journal.pone.0018379)
Supplement: Table S2 — (DOC) [file pone.0018379.s006.doc]

**Table S2 Real-time PCR confirmation of sex differential expressed genes predicted by Poisson-based enrichment test and based on annotation (successfully validated)**

| contig ID | #female reads * | #male reads * | Primer ID** | Ovary verse Testis | | Female Liver verse Male Liver | | Seq.  Length | Seq.  Description | min. eValue |
| --- | --- | --- | --- | --- | --- | --- | --- | --- | --- | --- |
|  |  |  |  | Fold | P value | Fold | P value |  |  |  |
| contig00138 | 738 |  | F1 | 1.84±0.54 | 0.0019 | 7.36 ± 1.84 | 0.007 | 1733 | vitellogenin a | 0 |
| contig44670 | 268 |  | F2 | 1.38 ± 0.35 | 0.0108 | 6.62 ± 1.71 | 0.008 | 894 | vitellogenin c | 4.13E-15 |
| contig01936 | 242 |  | F3 | No difference |  | 6.67 ± 1.32 | 0.004 | 1515 | vitellogenin b | 0 |
| contig02181 | 117 |  | F14 | 1.97 ± 0.61 | 0.0231 | No difference |  | 594 | ras gtpase | 0.10 |
| contig01827 | 110 |  | F17 | 10.31 ± 1.21 | 0.0004 | No difference |  | 1308 | b-cell translocation gene 4 | 2.35E-71 |
| contig40031 | 106 |  | F19 | 1.62 ± 0.62 | 0.0475 | No difference |  | 1012 | novel protein | 3.47E-34 |
| contig07134 | 105 |  | F20 | 3.06 ± 0.89 | 0.018 | No difference |  | 597 | transmembrane emp24 protein transport domain containing 5 | 1.46E-17 |
| contig01721 | 100 |  | F22 | 6.31 ± 1.19 | 0.0019 | No difference |  | 649 | ---NA--- |  |
| contig02933 |  |  | G9 | 2.41 ± 0.79 | 0.0286 | No difference |  | 259 | daz associated protein 1 | 0.68 |
| contig03679 |  |  | G16 | 2.11 ± 0.69 | 0.0286 | No difference |  | 1724 | sperm adhesion molecule 1 | 5.14E-12 |
| contig10040 |  |  | G11 | 8.69 ± 0.10 | 0.0003 | No difference |  | 417 | si:dkeyp- protein | 8.58292E-21 |
| contig02201 |  | 179 | M16 | -6.23 ± 0.72 | 0.0001 | No difference |  | 1612 | methyltransferase family | 3.81311E-37 |
| contig30427 |  | 173 | M17 | -7.42 ± 0.82 | 0.0001 | No difference |  | 377 | ---NA--- |  |
| contig30428 |  | 142 | M22 | -4.27 ± 0.94 | 0.0039 | No difference |  | 980 | coiled-coil domain containing 39 | 7.34E-05 |
| contig20381 |  | 134 | M21 | -7.12 ± 0.49 | 0.0001 | No difference |  | 285 | c-type mbl-2 protein | 0.21 |
| contig32045 |  | 134 | M12 | 1.82 ± 0.63 | 0.0337 | No difference |  | 896 | ---NA--- |  |
| contig30599 |  | 130 | M14 | -5.74 ± 0.65 | 0.0001 | No difference |  | 592 | zinc finger protein 329 | 0.27 |
| contig35727 |  | 116 | M23 | -4.51 ± 1.13 | 0.0071 | No difference |  | 625 | ---NA--- |  |
| contig33067 |  | 112 | M13 | -6.37 ± 0.79 | 0.0005 | No difference |  | 902 | solute carrier family 1 (glial high affinity glutamate transporter) member 2 | 4.95E-26 |
| contig45247 |  | 106 | M7 | -6.32 ± 0.65 | 0.0001 | No difference |  | 316 | type-2 ice-structuring protein precursor | 2.93E-15 |
| contig22779 |  |  | G20 | 2.39 ± 0.86 | 0.0387 | No difference |  | 333 | spermatogenesis associated 13 | 1.14E-16 |
| contig30745 |  |  | G29 | -4.70 ± 0.33 | P<0.0001 | No difference |  | 528 | synaptonemal complex central element protein 2 | 1.47E-29 |
| contig02487 |  |  | G23 | 3.04 ± 0.83 | 0.0144 | No difference |  | 1439 | serpin peptidase clade b member 1 | 2.96E-163 |
| contig00883 |  |  | G21 | 3.35 ± 0.89 | 0.0134 | No difference |  | 207 | spermatogenesis associated 5-like 1 | 0.12 |
| contig04624 |  |  | G15 | 3.92 ± 1.01 | 0.0114 | No difference |  | 741 | sperm acrosomal membrane protein 14 | 0.14 |
| contig05309 |  |  | G13 | -4.56 ± 0.87 | 0.0033 | No difference |  | 699 | ovary-specific c1q-like factor | 0.16 |
| contig06275 |  |  | G26 | 3.52 ± 1.08 | 0.0221 | No difference |  | 1159 | ---NA--- |  |
| contig06905 |  |  | G6 | 4.93 ± 0.96 | 0.0037 | No difference |  | 706 | nuclear autoantigenic sperm protein (histone-binding) | 7.84E-78 |
| contig07643 |  |  | G34 | 2.73 ± 0.79 | 0.0185 | No difference |  | 397 | four and a half lim domains 3 | 1.50E-25 |
| contig07774 |  |  | G31 | 1.88 ± 0.66 | 0.0361 | No difference |  | 1123 | sry (sex determining region y)-box 5 | 1.32E-16 |
| contig14446 |  |  | G30 | 4.67 ± 1.04 | 0.0064 | No difference |  | 441 | sry (sex determining region y)-box 4 | 1.86E-03 |
| contig15766 |  |  | G8 | 6.98 ± 0.96 | 0.0008 | No difference |  | 791 | bcl2-related ovarian killer | 3.82E-82 |
| contig30647 |  |  | G28 | -5.49 ± 0.41 | P<0.0001 | No difference |  | 682 | sperm autoantigenic protein 17 | 7.34E-03 |
| contig26089 |  |  | G2 | 2.16 ± 0.66 | 0.0218 | No difference |  | 251 | sex-determining protein dmo | 4.77E-15 |

* Enriched with significance 0.001

**F and M indicate genes predicted by Poisson-based enrichment test. G indicates genes predicted based on annotation results.
