# Supplementary material for: Transcriptome Analysis of Female and Male Xiphophorus maculatus Jp 163 A
Source: PLoS One. 2011 Apr 5;6(4):e18379. doi: 10.1371/journal.pone.0018379 (PMC3071723; doi:10.1371/journal.pone.0018379)
Supplement: Table S3 — (DOC) [file pone.0018379.s007.doc]

**Table S3 Real-time PCR confirmation of sex differential expressed genes predicted by Poisson-based enrichment test and based on annotation (Unvalidated)**

| contig ID | #female reads * | #male reads * | Primer ID** | Ovary verse Testis | | Female Liver verse Male Liver | | Seq. Length | Seq. Description | min. eValue |
| --- | --- | --- | --- | --- | --- | --- | --- | --- | --- | --- |
|  |  |  |  | Fold | P value | Fold | P value |  |  |  |
| contig39984 | 188 |  | F4 | No difference |  | No difference |  | 971 | myosin heavy chain | 2.47E-84 |
| contig01720 | 182 |  | F5 | No difference |  | No difference |  | 358 | ---NA--- |  |
| contig45262 | 162 |  | F6 | low expression |  | low expression |  | 229 | ---NA--- |  |
| contig00069 | 159 |  | F7 | No difference |  | No difference |  | 353 | ---NA--- |  |
| contig08354 | 148 |  | F8 | No difference |  | No difference |  | 406 | ---NA--- |  |
| contig44129 | 138 |  | F9 | No difference |  | -1.05± 0.12 | 0.0001 | 341 | protein | 6.44E-36 |
| contig43740 | 134 |  | F10 | No difference |  | No difference |  | 543 | b chain rhamnose-binding lectin csl3 | 0.72 |
| contig02424 | 127 |  | F11 | No difference |  | No difference |  | 340 | ---NA--- |  |
| contig43726 | 125 |  | F12 | low expression |  | low expression |  | 225 | ---NA--- |  |
| contig01883 | 116 |  | F15 | No difference |  | No difference |  | 556 | zgc:158463 protein | 1.20E-62 |
| contig01475 | 112 |  | F16 | low expression |  | low expression |  | 361 | ---NA--- |  |
| contig00531 | 108 |  | F18 | No difference |  | No difference |  | 176 | myosin heavy chain | 1.35E-17 |
| contig43741 | 105 |  | F21 | No difference |  | No difference |  | 245 | ---NA--- |  |
| contig20152 |  |  | G35 | No difference |  | No difference |  | 638 | lim homeobox 2 | 1.48E-16 |
| contig20615 |  |  | G10 | No difference |  | No difference |  | 274 | gpbp-interacting protein 130b | 0. 70 |
| contig43161 |  |  | G32 | low expression |  | low expression |  | 317 | cation sperm associated 3 | 7.33E-25 |
| contig45318 |  | 889 | M6 | No difference |  | No difference |  | 609 | atp synthase f0 subunit 6 | 1.75E-71 |
| contig45477 |  | 675 | M19 | low expression |  | low expression |  | 149 | ---NA--- |  |
| contig45523 |  | 627 | M3 | No difference |  | low expression |  | 281 | ---NA--- |  |
| contig45523 |  | 627 | M8 | No difference |  | No difference |  | 281 | ---NA--- |  |
| contig45522 |  | 548 | M4 | No difference |  | No difference |  | 280 | ---NA--- |  |
| contig40414 |  | 267 | M2 | No difference |  | No difference |  | 919 | ---NA--- |  |
| contig32045 |  | 134 | M11 | No difference |  | No difference |  | 896 | ---NA--- |  |
| contig41732 |  | 129 | M1 | No difference |  | No difference |  | 1246 | cytochrome c oxidase subunit i |  |
| contig30607 |  | 121 | M10 | No difference |  | No difference |  | 867 | early b-cell factor 2 | 0.12 |
| contig45151 |  | 116 | M15 | No difference |  | No difference |  | 159 | ---NA--- |  |
| contig40011 |  | 111 | M26 | No difference |  | No difference |  | 1012 | ---NA--- |  |
| contig11497 |  | 106 | M24 | No difference |  | No difference |  | 577 | ---NA--- |  |
| contig30170 |  | 104 | M9 | No difference |  | No difference |  | 391 | ---NA--- |  |
| contig30184 |  | 100 | M25 | No difference |  | No difference |  | 774 | ---NA--- |  |
| contig30993 |  | 100 | M18 | No difference |  | No difference |  | 952 | intraflagellar transport 172 homolog | 2.99E-13 |
| contig00824 |  |  | G24 | No difference |  | No difference |  | 480 | sex hormone binding globulin | 1.28E-48 |
| contig01422 |  |  | G3 | No difference |  | No difference |  | 1407 | isovaleryl coenzyme a dehydrogenase | 1.19E-168 |
| contig01592 |  |  | G12 | No difference |  | No difference |  | 713 | complement component q subcomponent-like 4 | 3.63E-38 |
| contig02196 |  |  | G5 | No difference |  | No difference |  | 662 | ---NA--- |  |
| contig05040 |  |  | G7 | low expression |  | low expression |  | 379 | ---NA--- |  |
| contig09487 |  |  | G14 | low expression |  | low expression |  | 474 | sperm associated antigen 16 | 6.48E-02 |
| contig13920 |  |  | G17 | No difference |  | No difference |  | 594 | sperm associated antigen 1 | 1.57E-17 |
| contig14696 |  |  | G19 | No difference |  | No difference |  | 960 | neurogranin | 0.28 |
| contig18544 |  |  | G25 | low expression |  | low expression |  | 514 | sperm associated antigen 11 isoform e precursor | 1.89E-35 |
| contig20031 |  |  | G1 | No difference |  | low expression |  | 258 | degenerative spermatocyte homolog lipid desaturase | 2.95E-28 |
| contig22887 |  |  | G4 | No difference |  | No difference |  | 345 | fatty acyl reductase 1 | 1.51E-32 |
| contig24990 |  |  | G18 | No difference |  | No difference |  | 574 | sperm associated antigen 9 isoform 1 | 1.49E-71 |
| contig27348 |  |  | G27 | low expression |  | low expression |  | 278 | spanx-c | 1.22E-26 |
| contig27967 |  |  | G22 | low expression |  | low expression |  | 457 | serpin peptidase clade b member 6 | 2.86E-68 |

* Enriched with significance 0.001

**F and M indicate genes predicted by Poisson-based enrichment test. G indicates genes predicted based on annotation results.
