# Supplementary material for: Transcriptome Analysis of Female and Male Xiphophorus maculatus Jp 163 A
Source: PLoS One. 2011 Apr 5;6(4):e18379. doi: 10.1371/journal.pone.0018379 (PMC3071723; doi:10.1371/journal.pone.0018379)
Supplement: Text S1 — (DOC) [file pone.0018379.s009.doc]

Complete list of contigs full sequences:

>contig00069

AGTGCggAGCGAGgAGACGCGCTgCATACCAACAGAGACACAAACGgAGAGGCTCGAGGT

TTTTTGGCCAGGACGCACAGACTCCATCCTGCTCTCTGAGAAAAACATTATCAAATCAGA

TTATCGGACGGTGTGAATGTTAACCTTTCAGTTTTCATTCGTTTTTAGAGAAATTCTGCT

TGTTCTTCAAGGCACTGAGAGCCAAACTAAGTCTGTCTGGGATATTTTTGCTTTCTGCTG

TAATATTTTTTtCTGGCAGACAGAGACATTTAAGAAAATCTCACTCTTTCTTTTTCCCAT

CTTTTGCTCTCTGTCTCTCTCTTTCTCTCTCTGACGGgACTTaaTtAAAAAAA

>contig00138

ttttttGAATTTTAAGATGCAGTTTATTTATTGTTACAGAAAAAATTTACACATAGTGAC

TTAAAGGAAATGTAGTTAAGAGCACTTGGTGTTGCAGTTGCAAGCCAGGTGAGCTTGGGT

AGTCTTTCTCAGGTCCACACTTCTGTCATAGACATTGCTCTCAGAATCAGATGACCAGCA

GTTGAAACCAACAGTGACGGGGGTGGTCTTGATTGGCAAGCATCCAGGCAGACAGCGAGG

TACAGGCTCAACAGAGTAGCATGTGGAATCGTCACCATGAACGGTCAATTGTTTTTCCAG

CTGCACAGATTCAAGCTTCAGACGGCACTCAGAGGCATCCCTGCAGCTTTCAACAGGCAG

AATCCAGGAGTGAGCAAAGCTGACTGAGTTCTTAGCCACGCGTCCGTTGGGAGTGTGGTA

CTCCTGTCTGATTTCTCCATCAGCCTTTCCACAAAGTCCACAGGTCTTTCCTTTCATCCA

GTCAGCAATTTTGATCCTCCATGTCTTTTTGTCAAAGTAGACTTCTTGGAGACCATGTTT

AGGTGCAAATACAGCAAGGCCCTCTCCACTCTGTCTGATCTCAATGGAAGCTGTTGGGTG

GCGGTAAGGCAGGCTGGTGAGGGGAATTTCCATTTCGTTGACCTTCACAATGATGTTGTT

GTCCCTTGGATACATGTCGATGTCAATCTCAGAAATTTTGACATTGATATGGTGTTGCTC

TGAAGAATCTTTCCTCAGGAGAACCATAAACTTCAGTTCATCAGTGCAGTCCTGTGCGGC

AACCTGGTAGCAAGAGGAAGGCATCTTGTTCTTGTAGCTCTTGTTGTTGAATGTGTAGAG

TGTGTCTTCAAAGAAGCTGCATTCAGCTGCGGCAGCCTTTGAAACCATGAAGTGGATCTT

GTCAATGACTTCATCAAATGGGGTGAGACCTTTGATCTCTTCAATGGGCATAGACATGGG

AAGGCGCACAGTGACATTGTAGACAGATTTCATTGGAGTTTTCACAATGAAGTCAATTGT

CCTGTCAGAAGTTGCAACTGCAATGACTGAGATTTTCCTGGTGCTGTTTTTTTtCTTTGT

GTGAATCAAGTCTGACAGTACTTTGGAATGGACATATTTGTTGACCATCTTTCCATAACG

TTTCAGGGCAGAAGGCAATCTTTCCCAGGACGCTCTGAGGCGAGCTGCCGGGCTTGAAGA

AACAAGACCAGTCTCTCCTGTAGCATTGGCGCTATATCTCTTGCATTGCTCGCCCCAGGC

AACCCTAGTTGTAACTTTGTGCTTGCTCAACACAACTCCATCAGCACAGAGCCTCCAGTT

GCTATCAGCAGAcAGGTtGGAGACAATGATCTGAAGTCTGGCGTTTGGTTTGTCAAGGTA

GGCAGCAAGTTGGTATCCCAACATCcTCTTGTCAACTTTGACAGCACGGACGATCAATGC

CACAACAATTTCCTCTTCccTGAGGAATTTTTtCTGATTATAGATGGCCTCAAAGCTGGA

GGAAGTGCTCCTGCTTCTGGAAGTTGATTTggAGGgATTCTCcTTATTGTGTAACCTCTG

GAACTTCTCAGTCACTTGCTTAGAGCGAGAGGAGCTGGAACGAGAAGAGCTTGAACTGCC

GCTGAAGAGAGATGCGATGCTTGaTGCAGAGCTGGTCCTATCTGAaCTGCTGCTGGaTCT

TGATAAGTTGCCGCTCTTGCTTCTGCGGCTGCTTCTCCTGCTTCTGCTGCTTc

>contig00531

tttttGACTCTtGGGtaGCACAGAGCCTTTAGCATATCAGCTGAGTTCAGACCCAgAAGG

TAGGCGATTTtATCAGCCACCTCAGTGCCATCTGGTTCAGCCTGCTCCTCACGCTGCTTC

TGCTTGAACTTCATGTTGCCATGATGCATCACAGCACCAGTGAGCTTGTAGATGTT

>contig00824

ACGACGGGAAGTGGCACACGTTGGATGTtGtAGCAACCAGGGAAAGTTtGtCATtCtGGA

GGTGGATGGCCTACCTGGACTGGTGGTAGGAATGCACTCCAAACAGACAAAGGAGGTCAT

TTCTGGGCAACTTCGATtGGCCCTTGGTGGGATCCTGACCAGCAAGGACAATTTGATCGT

TGAGTTTGAGCCACAGATGGACGGCTGCGTGCGCAAAGGCCACTGGCTAAACCTCAGCAT

GCCCTGGGAGGCGGACGCTGACGAGCTCTGGCTCTGCCATCAGAACATCCAACCTGGCAG

CTTTTTCGCTGGCGAAGGATTCACCATTTTCAACACCTCAGTTTTCCAAACAATCAAAGA

TCTTGGCTTCAGAGTTGAATTTTGGGGGgACTTCACTCAGATGGAGGgAACCATTCTGAG

CATGATGTCCTCACAAGGAGAGCTGCTGGCAGTCCTGGAAGCCAATAATAACACgAATGA

>contig00883

TCACATGTTGCTGACATCACACAATTtCTCATCTTCACACCCTGACTTTAATAATAAGCT

GGGTTTATTATTTTATTTTATTTATTTATTTATTTTAACAGCTGGACTGTCAGGAGGTTT

GTAACAAAGATGTGATGGTTGTAGCTGCCACAAACAGACCCAGCTGTATCGACAGCGCCC

TCCTCCGACCCGGCAGGCTGGACCACa

>contig01422

aaCGGGACTGGGTACCTGGACCACGTCATCGTCATGGAGGAAATGTCTCGGGTGTCGGCA

GCCATCGCTCTCAGTTATGGCGCCCACTCCAATCTCTGtGTCAACCAGaTGGTGCGTCAC

GCCAACCCGAaGCAGAAGGAGAAGTACATGCCAAAGTTGCTGACAGGTGAACACGTTGGA

GCGCTGGCCATGAGCGAACCTAaCGCTGGATCCGACGTCGTCTCCATGAAACTcaaaGCT

GTTAAGAAaGGTGACTATTACGTTCTAAACGGCAACAAGTTCTGGATCACCAACGGGCCG

GATGCCGATGTCGTCATCGTGTACGCCAAGACCGACCCGGAGGCGCATCAGAAGGGAATC

ACGGCTTTTATTGTGGAAAAGGGAATGCCAGGATTTTCCACTGCGCAGAAACTCGACAAA

CTGGGAATGAGGGGATCCAGCACCTGTGAACTGATCTTTGAAGATTGTAAAATCCCAGAG

AAGAACATTCTGGGTTCGCTGAATAAAGGAGTTTATGTGATGATGAGCGGCTTAGACATG

GAGAGGCTGGTGTTGGCTGCTGGACCTGTTGGCATAATGCAGTCCGTTCtGgATGCTGCG

ATCCCCTACTTACATGTGAGAGAAGCATTTGGGCAGAAGATTGGACACTTTCAGCTGATG

CAGGGCAAGATGGCCGACATGTACACCAGACTGAGCTCCTGTCGGCAGTACCTCTACAAC

GTCGCCAGAGCTTGTGACAAAGGGCATTTCAGTGCAAAGGATTGTGCTGGAGTGATTCTG

TACTGCGCCGAGAATGCCACTCAGGTTGCCTTGGATGGCATTCAGTGTTTAGGCGGGAAC

GGCTACATCAACGACTATCCGATGGGGAGATTCCTGCGAGACGCGAAGCTGTACGAAATC

GGGgCGGGAACCAGCGAAGTCCGCCGGATCATCATCGGCCGAGCCTTCAACTCCATGTTC

AGATAGACGCAGCAGTCGCTCGGTTTGTtGTTTGCATCGTTTCTCACGCCCAGGACCTTA

CTGGATGAGTTGTTGTTGTTTCCATTCAGACGATAAGCTGATAAATGAACAAATTACATA

TTGATCCTCGCCGAGCGGCCTTGAAGTTTTGCTATGCTAGCTGCCTGTTAGCTGGAGGGA

GATGATAATAATGTAATGAACAGATTCCCACATTTCAAGCTTAAACTCTTACTCAAAACA

ATTTGGATAAAATCATCTTTAATAGATTATTGAAGGTTTTCCCTAAGCTGATTCTGTTAT

TGAATATTTTTCTGTAAACAAAACACTGTCAAACATATTTTACTTTTTtGTCTGTGAAGA

TTTTGTCTCTTCTTGATTGTAGGTGTCATCATTATGTAGAAGTGTATGAACTATACCAGG

TTTGCAATAAATCAATAAGTTTCTAAt

>contig01475

CAttGCTTTTGCAAAAGAGTTCCCTAAAAAaTAACAAAACAAAACAAAACAAATTTGGAG

AATTAACCTGTTCTAGCAGAGAAGGATCATAAAAGAAAAGGTAAAATGAGAACAAAATTA

GCTTCTAATAGAAACCCCACACATCCTTCTCTTTTCTTGGATTTTACCCTATGAAACCAC

CTGCAGCTCCTAATAGGCTCATATATAAGGTGTCAAGGTGTGATAAAATACCTTTTCAGA

TAAATTACTGGCCTTAAGAGGACTTTTTTTTtGAAATGTTTTCCCACCTTGTGTCTCTAG

CTTGGaTTATTCTTGGACACATGTAATTAATTGGAGGGGCACTCCTAaGTaGGcaCAACa

a

>contig01592

GCggggACAGTGTTGCTTGTtCTGCAGCAGGTGAAGTTTTCCTAAAGCAAAGATGAGGTC

CTtCTTTTTTCTTGGGgCATtGTtGGTTtGCGGTTTGGTTGGATCCCAGGATACCATACA

AAACCAAATCCAGCAGGTCATGGCCAGACTGGAGAAAATTGAGAGGGAGAACGAGGAGAG

AGCTAACAGGGCTCAAGTGGCATTCTCTGCTGCACTCGTCGAATCGCAGGACTGGACCTC

CATAGGGCCTTTTGATCGTTGTCACACACTGGAGTTCAAGAAAGTGATAACGAACATTGG

CAATGCGTATAATCCAGAGACAGGAATTTTCACAGCCCCAGTGAAAGGACTCTACTACTT

CCGGTTCACCGGAGTAGTTGGAGAGACAGGGAAACTGAACGCAGGACTGAAGAAAAACGG

TGAGAACATTGTCGCAATCTACCACAAAGCAGGGAGACAAGCCAGCGCTTCCAACGGAGT

GGCGCTGGAGCTGGAGGAGGGGAACCAGGTCTACGTCCAGCTCTTCGAGAACGAACTGAC

CATTGCAGATCAAAACCGGCTCAGCACCTTCAGCGGGTTCCTTGTCTTCCCTCTTTGAGG

GAAACCTCTGACCACCAAAACGCAAAATGCATGATGTCATTTCTTGAATGTTTTGTGTAA

CTGAGCTGAAAACTaCAAACATAACTAAATAAAAaCaTAACACTcTaaaaaaa

>contig01720

ttCTAATCGCTCAGCATTTGGAAGCGTGGAAAacaGGGACTGAAGGGCTTCGTtCACTTC

CTCCACTGCCGTTGCTAAAaCaTCACAGAAAaTAAACGGCAAGCaGtCACAACTTCTCCT

GCCGTTCACTGATTGGCTCGACTGAAATTCTACCGGACAAATCGAGTTCAATGGCAGAAA

GCCCAGATGGAGCACTGAAGAGATGAGAATCGAGCAGAATTGTATAGGAAGGCAATGGAC

ATTCTATATGTTTCTCAAATACTGCTAAAATGAGGCTGGGTAAAAACTGTTTATGTTTTt

CAAAGAGGTACAAAATGATCCCACTGATTGGGAGAATCTTATAAAGACATAAATAAGT

>contig01721

aGTGATTTAAGAAaCTAAAATATTACTTTGAAtGAACAATTTACTCCcTTATTTGGCACA

GAtGAATTTAAGAAAAGGTTTATTTTTTtACTTTTAATTCATAAATGTATACAGACAATG

AAAATGAACCCCTCTTATCATTTCCAGAGAAAAGCCTAAAAGTTAAAGTATGTCCATCAC

ATCCTACTTAATAGAACAGCTTTTTTCATGCATAGATGAATTAAAGTTGCAGGCAGCAGC

TACAGAAAGCCGAGTCGTGATTCTCCTCCGATTCTCCGTCACCATGTTGGTTCATTGATT

ATATGCACGACTAAGTGATTCAATCATGTACGTGAAAGAGTGGCCCAGGAATACAAGGAA

ACCAAGCAAAGGCGGGATAAACAATAATAAGTGGCAGCAAAAGAAGCCAGCGCTGCTTTT

ATGACTCGCAAATCTCCAGGTTTCAAAAACGATTCTCCGCAAAATGAAGAACATGCCCAA

GAATGAGAGAGTGAGGAGAGTGAAAAGGACAATAAAGTGAATCCAGAAGTTGTCCATTGC

AGAAGCTGCAGGTGTCATCGTAGTAGGCTTAGTTCAGAGTTTGCCTGTATACAAAAAAaG

TTTCCTTGTCAGTGAGCTGCAGGAAAaTGTTTCTCTTTCCCGTCCCCGc

>contig01827

gAtGATGCAGGAATAAATTtATTTTtCAATGACAGTTCATTCTCACTTTATTCACACTTG

ATTTAACCTCAGTTAAAAACACATAGAATTACATTAAAATAGCACAGTACGTTTCATAAA

AACCTGCTTAAACAGCAATACTTTAAACTATCAATTTAAACCAATTCAAATAAACTAATT

TAAAGATTCAAACACTGTGAAAAAGTCTAGTCTTAAAGCAATACATTAAGTACTAAGGGA

AGAGCCACTGCCAACTTCAAGATCGAGATTTGGAGACCCAGTGGTACTTGTCGACACGGG

GCCCCGCAAAAGTAAACGTGGTCCGATTGGATTTAAAGCCTTTCTGGTTGGAGAACTGAG

ACGGTGGACATCCAGTGGAGGAAGAGAAAGACTCTCCAGGGAAACCCTTCCTCTTTTCTC

TCAGCCAGGTCTGTGGAGCTCCTGGAGAGAACTCGCTTAACcGGTAAATGCTGTTGGGGT

TGATGACAGTTGGGATGGTTTTAGGCTCGAGGCCTGCGGAAGGGCAGGGGCCTGATGGAC

GGCGGCTACTGTTGCTACTCATGCTGTTGTCcACATCTTTGTCTTCTTCATCaGAAGAAC

TTCCCGACTGGATGTCGAGGCTTGCTCGCTCCACAGCCTCATGGATGCGTCGGGAAAATT

CACCATCGCGCCGACAGCTGTCCAACACCGAGACGCAGAATGGAGTgCTACGTTCACCAT

ACCTGCAAGAAACCTCTCCAGGGTCGACCCACACTGTGATCTCCAGGGGCAGCCCCAGGT

CTTCATACCGCACTGCGCTCCACTCACAGGCCTGCTGCAACACCGGGTCCTGCAGCTGGA

CACGGTTCATACGAAGACACCTGTAGGCCTGTCCTTTGGTGGGCACGCTTGGGTGCCAGT

GATTCTTGTAGTtCTCGAaCAGAGCCGAGGTGAGGGCGGCAGCAAAGCGCTCCCTGCTGT

CGTTATCCAGACAGCCGTAtCTTTTtACCAGCCGAGTaGTAAAAAaCACAGCGGCAGCAA

TTTCCTCTTTCATGGCTTACACACTCTACTTTCCAGCACAAAACACAGAAGCTATTCAGC

TAAATAAGAaTAAAAAaCAAACTCAGATCATAAAAAGACTTGTACAATAAGTCCACTTTT

AAAGAAATAGAAATATAAAATTAACCAGACAGAGACAACGCAAGCAAATGTATTTGCGGC

AAAAATTTCATTAAGCTCTCTGGATGGGCTACAAAATCTAAAAAACATAAATCGCAACAA

ACTTAACTAGTCAAAAGCAGCAAGATATCAGCCTCAACACTTTaCaaa

>contig01883

GGACACGGAAAGGATTGACAGATTGAttAGCTCTTTCTCGATTCTGTGGGTGGTGGTGCA

TGGCCGTTCTTAGTTGGTggAGCGATTTGTCTGGTTAATTCCGATAACGAACGAGACTCC

GGCATGCTAACTAGTTACGCggCCCCCGTGCGggTCGGCGTCCAACTTCTTAGAGGgACA

AGTGGCGTTCAGCCACACGAGATTGAGCAATAACAGGTCTGTGATGCCCTTAGATGTCCG

GGGCTGCACGCGCGCCACACTGAGCGGATCAGCGTGTGTCTACCCTTCGCCGAGAGGCGC

GGGTAACCCGCTGAACCCCACTCGTGATAGGGATTGGGGATTGCAATTGTTTCCCATCAA

CGAGGAATTCCCAGTAAGCGCGGGTCACAAGCTCGCGTTGATTAAGTCCCTGCCCTTTGT

ACACACCGCCCGTCGCTACTACCGATTGGATGGTTTAGTGAGGTCCTCGGATCGGCCCCG

CCGgGGGTCGGCCACGGCCCCTGGCGGAGCGCCGAGAAGACGATCAAACTTGActATCTA

GAGGAAGTAAAaGTCg

>contig01936

ggATTTCTCACtCTTTTTATTATTAATAATaCATACAATTAAGAATGCAATGAATGATCA

ATGTTCTTAACCCAACATTTATTTTtAATTATACCTTTTATTACTTTGTGCCATCACCGT

TCACCACAGAAAtAATTTCAATTGCTTCTTTATTTCAGTTTACAAGGATCCAGGAAACAC

AAAAACAGAACGAATGTCAGAAAGCAACACAATTAGGCACACTGTGGAGTGCAGCGACAG

GCCAGGTGGGACTCCGCTGTCTCGCGCACATCAATGCTCTTCTCGTAGATGCTACTGAGG

CCATCAGAGCGATTCATGTTCGTCTCCATAGGAACGCAGTGGTAGCCGACAGTGACGGAG

GTGGTTCTCACTGGCTGGCAGCCAGTGAGACAGCGCAGCACAGGTTCGACAGAGTAGCAT

TTAGATTCCTCACCTTCTACCCTGACCTGCTTCTCCAGCTTCACAGATTCAAGCTGCATG

AAGCACCCAGAGGCCTCACGGCAGCTCTTTGCAGGCAGAACCCAGGAATGAGCGAAGCTC

GTTGCGTTCCTGGAAACCCGTTCGTTCGGAGTGACATACTCCTGTCTGACTTCACCGTCA

GCTTTTCCACAGATTCCACAAGTCTGGCCTCTCATCCAGTCCACCACCAAAACCTGCACT

TTATCAGAGCTGAAGAAGACTTCCTGAAGACCATGACTGGGACCAAACAGAGAAATGCCC

TCCTCCTTCTCTTTGATATGTATTCTGCCTGATGGGTGCCGATATGGCAAGTTGTTGAGA

GGAATTTCTGCTCCATTAACCTTCACCATAACGACTTTGTCCTTGAGATACATGTCAACA

TCAATGTTTTCAATCTTGATGCTGATCTCATTCTGTTCTGCTGTTTGATCCctCTTCAGC

AGAACTATAAATTTGAGTTCATTTGTGCAATCTTGAGCCAAGACCTGAGGGCAAGAGTGG

GGCATCTCTGTTTTGAACTTCCTGTTGTTGAAGGTCACCACTGCGTCATTGACCACGGCG

CACTCAGCTGCGTTAGACTTGGTGAGCATGTAGGTGACCTGCTCCATCCAATTGCCCTGA

TATGCCTGCATCTCAGCAGCAGTGTTGTTCATCGGCAGGTATAAGGGAGTGACCCATCCC

AGTTTGTAATAGTTGCTCTTTGGTGTCTTCAGTGTGACATTCAGGCTTGTCTCGTTGACA

ACAGCTAcAGTGAGTTTTATCTGTCTGGGTTCATTGAGGGCCTTtGTCCTGTTCACTCCA

ATGTCCTCAGCGACACGGTAAAACCAATTAGAGACTCTTCTTGAATATTTCTTCATGTAA

TGAGGGAGTCTCGTCCAGGCCAGTTTcACACGGATTGCAGGTTTCTTATCAACGCTaCCG

GTTTCAACTGTGACCGTGGTGTTGAACTGCTTGCATTCAGCTCCCcAGGTTAGTCGAGTC

ATCACTTTGTGGTAGCTGAGCATCACGCTGTCAGTACAGATCGTCCAGTTGTCATTTTCA

GTGAGGTTGGCCACa

>contig02181

ACTtCGTTTTTTtGtCAGGCATGACCGAGTACAAGCTTGTGGTGGTCGGTGCTGGAGGCG

TGGGGAAGAGCGCTCTCACCATCCAGCTGATCCAGAACCACTTCGTGGATGAATATGATC

CCACCATCGAGGTGAGAACAAGACCGTCACACTGCGATCACCCCTCCAGGAAGCTGGATT

TGCTCTAAATATCAGGGGTTATTCTGGTGTTAATGGCTGCAGGACTTTTAAAGGCCACTG

CAGTCTGGATCTGTCATGTTAGAGCTCATGTTGTTGGCACCTAAACTTAAGAGGACTGTG

TAACATAAAAGTTAAAAATGTAAATTAGAGTAGAGAAAAAGTGTTTTATTTATGTAAAAG

TAACTTATCTCGTTTCAGCTTCATTTCAATGTCATGTAGCTTCCCACTCCCAATTTTTAT

AGATCATTAATAATCATGTAAAAACGATTTTATTCAGATTAGTCACGGGCCCTTTTTGGG

ATTCAGAGTAGAAATGTACAATAGCATTTTCATGTAACTACTCCTCTAGGTTCTAATTTT

ATATCAATGCATTTGTCATTTTGATTTCGACATGTAGATTTAAGCaaaaaaaaa

>contig02196

CTGTGCCTGTCTACCTCCACTTAAGtGTCAACAAGAAACTGGTAGCTGCTTATGTTCTTG

GTCTTCTGACTATGGTCATCCTACGTACATGAAGTGCACTGCGCTGAAaGGACgAGGATA

AACCGTTTTAAGCGATGAAGAGACgTgAAGAGAAACGTGACAATGACTGTAACTGTGATT

ATCAACATCGTGGAACCAGAGGCACGACTTCAACGAGCAGAGCTCTCAGCGCTCTGGTGG

AgCTCCATAACTCCATCCGCTGACAAATACATTTtAGtACATtGGTTGCAAATATATAAA

TGTTTTCTGGGCTGTTaCAAAAAaCAAAAAAaCAAaCGGgAAGCTCTATGGGAACTCTTG

CTGAGCAGACGCcGCCTCCGAGCCTCGGCCAGGGAAGAGACGCGGGGCGCTTGGGACGTC

GCCTGGACCGTCATGCATTCTTCGTAATTTTATTGCCATTGTGTGACTCAATATGGTTAA

CTTGAGTGTGAAGAAATCTACATGAAAACGTGTACATAAAGCCATTAAAACGTCATGATA

TGCACCTgTAACTGTGGTCATATTGATATTTTGAGGCTTTCATAGCACAAGACTGAAGAC

GATGCATTCAAGTGCATGTGGCTGCTTCTGTCTCTCACGTAAaCCCTTGAGACACTTTTt

Ac

>contig02201

CGTGGTTTCTCTTCACAGATTCTCTGTGTGGTTTtGGTCATTAAAATCAGGTACAGGTAC

TTTTGAATTTGAACCATTAATCAACACTAATCAATCTGCTCCAGCCTTCTGCTTTATAAT

CCTCTCAAACCAGAaCTCATCCTTGCTGCTTGTTTCCCAACTCACATTTTCCTtCTGCAC

AACTTTCTATTGATATTCTTGGATGTATCACTCTGCAAACGACCTGCTTCTTTACCAGTG

ACCTTaGCATTGTGGACGGTGATACTGACAGAGTGTTGGACACAGTCAGCAGTTtCCCCC

ATGACTGTTTATTGtCGtAGGCCAGGTTAACAAGACACTCAAACTGAAGTGgCCATTTTG

CAACTTTACTTTTGTGTAAATATAGCTTAGCATACAAGTACAGAGGTTCTTCAAGTTAAA

TTCCTGCAGTTTGCAGTTTAAAAACTACAAACAACCCGAGCTCAAACTTGAAACAGGTTG

AGGATAGATTAAATGGGAGGTAATGCCTCATATTGGACGCTGACGAAGGGCGGCAACATT

ATCAAACTGTTGCTGAAAAAGTTGCCAGCGTTTCGAACCCAGCAGCCACAAAATATAAAG

ACCAAAACACACACGCTGAAGATACACAGCATGATCAGCTTCCGGATGGTGGCCCTCAAG

ATAACAGCACAGCTGCTCCCACGTTTGGGTTGGAGGTTAGTTGCAAGACGGGGAGGTTCC

AGCTGCTCCTTCTCACCAGTCTTTATATTGTCGTCCAGATCAGATGCcacAGAGaTCGGA

TGCATGGTGTCTTTtGTGCAGAAATTCATGCCAGCAGACGAGAAGAGGTGCTGGATTTCC

TCTGCTATGGACAGGTGAGGACTGGGCTGTCCCTCCTCCTCTCTCATTGCCTCAGTCACA

GCAGCAAATGAAGCCTCGTCTCGTCTCTCCTCCAGCTGGTGGATCAGCTCCTGCTTCGTC

AGCCTGAGGTCCTTCATGACTTCTGAATATTCCTCCACGGTTTCCTCCAAGTGCTTTAAC

TGCAAGGTTTTCTGATGAATCAGCTCATCCCTTCGCTGCAGCTCTAGTTGAACCTCCTCC

ACTCCAATCTCAAGGTTTTCAAATTCAGTCCTAAGCTTGTTTAAAGCGATCTTGTCTTGC

TCTTGTTCTTGTTTAAGGATCTTTATCTTGGTGATTAGCTCCTTATTTTCTTCCTGGACc

TTTAATGTTTCCTTCTCCAAATCCTGAATCTTCTTTTCACAGATTTTATTTTCATCCAGG

CCATCGGGCAACAGAGGCCCACAAGGTTCAACTTCAACCTGTTGAAGCTCGCTGAAGATC

CTCTCCAGGTCTTTCACTTGTTTCCTGAGGCTGGCATTCTCTGAGCGCAGCACTGCCATG

TCGTCGTCTGCAACATCCAGCCAGTGCCTCATCTCAGTGTTCAGTTCCCTCAGCTGAcTc

TGGCTGTAATCCAGCTCAGCTATTTTCTCCAGCATATCCATTCGGGATATATTTTCCTCT

GTAATGTCCATCGGGCTGGCCAAGTACTCCCCTGTGCAACAAGAACCTGGAAGCAACAGG

TGTGGCAGTTGTCAGGAACTTTTCCcAATGGCGGCCGTTGAAAGTCGGACtt

>contig02424

ttttttttCCCTAGTATGGTTgTTCACATTTCTATATACAACTTACATATATGTGATCTC

CTGTACGCGCTGCAGAAGACCTGAAAGTGTCCTGCATGCACAGTAGATGGCGTAGTAACG

TCACACACAGAGAGCATGCTCAGCGTTTTGCCAACCGCCATAAAGAAGAAGAAGAAGTGC

TCCGTGTTTGCGGAAGTAAACATGGATGCTAATGACCGGAGCTAGCTCATTAACAACTTA

ATCTTCATTATAGTGCGCTATGATTGTTGCTTTtCTtCCCGCTTGCTCTCATCAGGACGC

AGAATAGTGACGTTTGTTGAGTATCAATGaCGTTaCAAAG

>contig02487

GTAGTCTTAGTTTATACAATCCTTTACAGTTGTCCTAAAATACTTTtACAAGTTGCTTCC

AGGACATTTATATATATATTTCtATTTTTTtAAAtAGATGCTTTActCATTTTATAAGTA

AACCATCTTTAACTTGTGCTGTCTTAAAAGGTTTGTGAaGCAAAGCCCAACAACATCAGA

CATGGCAGTCAACAGCAACGCAGACTTTGCCCTTGGATTGTTCCGCATTCTGAGCCAAGC

AAATCCGGCCAGGAACATCTTTGTCTCTCCACTGAGCATCAGCTCGGCGATGGCAATGGT

CTATTTAGGTGCTAAAGGAGACACTGCAACTCAGATGGCAAAGTCCCTCTCATTCACTAG

GGGTGAAGGCGTCCATGCAGATTTTCAAAACCTAAACACTGACATAAACTCCCCGTCTGC

ATCGTACATCCTGAAGGTGGCCAACCGTCTTTATGGAGAGACCACGTCTAATTTTCTCCC

TAAATTCTTAGAAGACACACAAAAGTTCTACCAGGCAGACTTGAAGGCTGTTAATTTCAT

CGGAAACCCAGAGGACTGCAGAGCTGAGATCAACACCTGGGTGGAGGAGCAGACAGAAAA

CAAGATAAAAGATCTTCTGAAGCCAGGAACTGTTAACACCATGACAAGACTGGCTCTGGT

GAATGCCATCTACTTTAAAGGAAGCTGGATGAGTCGTTTTGATGCGGCCAACACCAAGGA

GATGCCCTTTAAAGTCAGCCTGAATGAGACCAAAACAGTCCAGATGATGTACCAGATGAA

GAAGCTGCCCTTCAACTACATCCCTGAGTTGGGACTGCAGATCCTTGAGCTTCCATACGT

GGAACAGGAGCTCAGCATGTTCATCCTTCTGCCACAGTTATCTAAAGATGGCTCAGACCC

TTTGCTGAAGCTGGAAAATGAGCTgACGCCAGAGAGGCTGGATGAATGgACCAACAGGGC

AAACATGGACATCCAAACAGAAATCATCGTCCACCTGCCAAAGTTCAAGCTGGAAGAGGA

TTACGAACTCAAGGAACATCTGTCCAAAATGGGCATGACGGACGTGTTCTGCGCTGGCAA

AGCTGACCTGACAGGCATGAACGAAGACGGGGGCCTCTTCTTGTCTACGGTGGCCCACAA

GGCCTTTGTGGATGTGAACGAAGAGGGCACAGAAGCTGCTGCAGCCACAGCTGGCATGGT

ATCTTTCTGCATGTTGAGGGAGGAGCACTTCCAAGCAGATCACCCCTTCCTCTTCTTCAT

CAGGCACAACAAGACCAAGTCCATACTCTTCTTTGGCAGGTTCTCATCTCCTCAAGAGAA

CTGATGTAATAAATTTGATGCTAAAATAAAAGCTGAAAAAGTTGTCATTTACTTTATCAA

ATAATCCTTTGCTTTTtGTTGACTTTAAGAAGTGATTACAATAAAAACATAGGAATgAt

>contig02933

ttttCCTTtAATTTTTTGTTTtGTTTTGTTTTACGTAGCAGTTTTAGAGGAGTTTTGCTA

AACCTCCTCCAGGTTCTTATTATCTTTCTGTTTTCAGGGTACGGTCAGGACCTGACAGCC

TTCGGCCACAGCTTTGTAGACCCCAGCCAGACGGCCGCCTCGTACGGAGCCCCTACAGCA

CAGCCGGCTGCCGCTCCCCAGCCCGCCGCCAGCGCGTTCGGCAGGGggCAGAACCACAAC

GTACAGGGCTTCCACCCCT

>contig03679

AATGCTccACTGACCAATGCAAGCAGCTTGAAATTCCACTGGATACTGCTGCCTTCCAGG

CAGTgacaacaccttccgctgtgccaggtcagtttcttaccatcttttatgaagatcgcc

ttggcctgtatcctaaagtcgACATAATCAAGCACAAGATCTACAAAGGTGGTATCCCcc

AAAACGGGAACTTGACAGAGCATCTTGCTAAGGCCAAACGTACAATAGATCACTATATCT

CCCAAGATTCCTCTCCTGGGCTGGCTGTGATCGACTGGGAATCCTGGCGCCCTCTGTggg

ACCAAAACTGGGGATCAAAACATATCTATCAGAAGCTATCTATTACTCATGCATTGCACC

TGGCTCCCTTTTTGACAACAAAGAAAATTTCCCAAACAGCCAAGAGCCAGTTTGAGCTAG

CTGGGAGACGCTTCATGGAAAAaaCTATCAGCATTGGCATCGGCAAACGTCCAAGCCGCC

GCTGGGGCTTCTATCTGCTtCCTGATTGCTTCAACTATGGATGGAACAAGCCAGGATACA

CAGGGAGGTGCtCcACTAAAGCtCAGAAGCAGAACAACAAGCTGCTGTGGCTGTGGGAAC

GAAGCACCGCCCTCTTTCCATCCGTCTACCTCCATATGACTTTGAGAAACTCGCCCCTGG

CCGCGCTCTATGTCCGTAATCGTGTCCAAGAGGCGCTGAGGGTGGCAGCGCTGCCAAAAC

ACCTCTACACTGCACCTGTCTACGTCTACTCCAGACCCCTGTACCGGgATCAGACCCAAA

TGTTTCAGACTCAGACAGATCTTGTTAACACCCTTGGAGAGTCTGCAGCTCTGGGTGCTT

CTGGGGTTGTAATCTGGGGAGGTACCAGGGACTACAACAGCAAGGCCTCCTGCCAGGCTC

TGTCTGAGTACCTGTCCTCCACGCTCAGTCCATATGTTGCCAATGTGACTGCGGCTGCCA

TGCTGTGCAGTAGGCTGCTGTGTAAGGGAAATGGCCGCTGTGTGAGGAAGAACTACAACA

CCGCTCACTACCTTCACCTGAACCCCTCCTCCTTCCGCATCCTAAAGGCAAGCGGGAAGT

ACGTTGCCGTTGGTCTCCCATCTGCCAGCGACCTCAGCAACTGGGTGGAGAACTTCACAT

GCCAGTGCTATGCTGGGTGGAGCTGCTTTCCTAAGCTGCGGCGTCCAACTCAAGTTCAAC

TTATAAGGGTTTAAATTGTGACGGGAGTAAATAAATGgCCCTTGCTGCCTTCTTGTGGTT

GTGTAGTCATTGCTGTGAGGAAATCATATCATAAGCAATGTTTGCTCACAAAATAAAGAT

ATgAGGCAAAACATTAAATTCCTGTTTCcTGCAATGGGATATTTTTCTTCCTTCACACAG

TTGCTGAAAATaaCTTTGTGTAGTTCAACACatttgcaatccagtgttggctgaaatgta

acaagaatgcttattttttCAGTttATTTTTCTAAGAATAAGTCACTGTCAGTCTCAAGA

CACTTTAATAGAAAAACTGTTCAGTTGTAAGTAATTATTTAATCAAATCAATACAACAGA

TTCtaattttattacatacaatcaaatgcaatcttaaatcaatgcagttgaattcagtta

attatgacaaatggttaaaaaatatattatatctgtccccctctgaccagtggCGTCAGG

GAGAAGATTGTGCAGCCTTTTGCCATTAAACAATTTATTGAATc

>contig03795

GTCGGGGACAGCCTCATCAGCcATGAAAGCGGTTGTACTAGCTCtGACTCTGGCCTTCGT

GGCTGGACAACATTTtGATTTGGTCCCTGAATTTGCTGCCGGTAAGACCTACGTGTACAA

GTATGAAGCATTACTCCTCAGCGGTCTTCCTGAGGAAGGTTTGgCAagaGCTGGACTGAA

AATCAGGTCCAAACTTCTCTTCGGCCGAGCTGACCAAAATaCTTtAATGCTGAAGCTTGT

GGAACCTGAGCTCTTTGAGTACAATGGCATTTGgCCAAACGACTCAGCAATCCCAGCAAC

CAAGTTGACGGCAGCCCTGGCACCTCAGTTTGCGATGCCCATCAAGTTCGAATACACCAA

TAGTGTTGTTGGTAAAATCTTTGCTCCTGAAGGGATTTCGCCTGTGGTGCTGAACATCCA

CAGAGGCATTCTGAATGTTCTCCAGCTGAACATCAAGAAGACCCACAaaGTCTTTGACTT

GCAGGAGGTTGgAACTCAGGGTGTGTGCAAGACCCTCTACTCCATCAGTGAAGATGCACG

TAATGAGAACATCCTTCTGACCAAGACCAGGGACCTGAACAACTGCCAGGAAAGACTCAT

TAAGGACATGGGATTGGCATACACTGAGAAGTGTGAAAAGTGCCAGGAGGAAACCAAAAA

CCTGAGAGGTACCACAACATTCAGCTACACCTTGAAACCAGTCGGTAATGCCATCATGAT

CCTGAAAGTGGACGTTAATGAGCTGAtCcAGTTCTTACCTTTCTCTGAGGATAATGGAGC

TACTCAAATGAGGACCAAGCAGTCCTTTGAGTTCCTTGAAATTCAGAAAGACCCCATTAC

ACCTATCAACGCTGTATATAAACACCGTGGATCTCTCAaGTaCGAGTTCTCCAaCGAACT

TCTTCAGACACCC

>contig04624

aTGGTGCTTTATTGACCAGCCTTGACCACAGTCAATCAGGAGGATGGATGAACAAGCCAA

CCTTTTCTCTCTGGGTTAAACATGTGAAAACCACCAATAACCAATACTATTTTTTCCTCT

TCCATATTTTCACATCAGTTTAAAACGTTTAAGCAAACAAACATCTGACTAAATGTGAGT

TTACTTTTGAGAACTAAGGTTAGGTCCAGCGGTAAACAGGCCTCCACTCCGTCTTCAACA

CACAGTTATAATTTTATATCagaaGATGATGCGGTTTGGTTTGAACAGGGACTCCGGAGC

ATCTTCATTAGAGTGGAGATGAGCTTATCTGGACTAGTTTTTAAATAAAGTGATGTGATC

AGGCTGGCGTTGGTCCTTGGTGTCAGGTTGCATTTGTTTCCACAGCAGCAGCTGTGAGTG

AGGTCGTACTGTTGACCCCGATATCTGACCATTTCCTGGGAGTTGCAGAGTTTGATATCC

AAGCAGCCTTGAGAGGACAGAACATGGCGGAAGCCTTGCCATCCCCAGGAACTGGCGCAG

CGATGGTTGGGCGGACACTCCGTGTTGAGGATTTGGCAGGGTACATTTTTGGGCATCAGG

GGGCAGAAGTTACAAAGAAGGGTCCCCACAGTGAAAGCAGGGAAGATCAAAGCAAAAAGC

AAGAGGGTAGAGGTCAGGTGCCAGGCAGAAACATGGACATCCACTCTGCTTTTTCTTTGG

TACCCCATCTTCCTGTCCCCG

>contig05309

aaactcaagtaaaacgctgcaggatcagatccaagctgtggcagcagaagtggctggtat

tagagccaaCACTGAAAACCAGGGAGATAATCTGAAGAGAGATGGAGAAGTGAAGATGAT

GGAGCTGCAATCCCTGAACCAACGATACCAAGCACAGGAATTTCAAGTAAAAGaCCTGCA

GGAACAGATCCAAGCTCAGGCAACAGAACTGGCTGCTGTTAAAGTCAACACTGAAAACCA

GTGGAGGCTCTGAAGAGAGATGGACAAGTTCAggCAGCAGAACTGGCTGCTGTTAAACTC

AACACTGAAAACCAAGTGGTTGCTCTAAAGAGAGAaGgAGAAGCTCAGGCAGCAGAACTG

GCTGCTGTTAAAGTCAAAGCCAACATGACTGGAAACCAAGTGGAGGCTCTGAAGATACAa

GgAGAAGCTCATGCAGCAGAACTGGCTGCAaTCAAGGCCACCATCAGTGAAAACCCGTGG

AGACTGTGAAGAGAAACGGAGAAGTCAAACGCCTGGCTTTCTCTGTCTCTTTGTTGGCAT

CGGATTATAGAACCATtGGACCaTTCAACACCGATATAACTCTGATTTtCAAAcgTGTTG

TTACTAATATTGGgAaTGCCTACAACCCagacACAGGACTGTTCACTGCACCAGTAAGAG

GAGTTTATCACTTTGAcTTCCACATAcATGGACATGGTT

>contig05040

cTTCGCCTCCGTGATCTTCAGGAtGtCAGGGAGCTGCTGCAGGTCAGCCAGCTGAGACTG

GAACTGCCTTCGTGCCGCCTCAaTCTCTTTGTTCATCTCATCTTTAAGGATCTTGTTCTC

TTTGTCACAGCGTTCTAGCTGGGCAGCTACTTCATCAGCCTCCAATCTGGTCTTCATCAC

CTGACTCTTATAGTTGTCAATCATCCCCTCATAGTTCTTCACTGAGGCCTTCAGCTGCTG

GACCTCGAGGTGTGCCTGGTTGAGTTTGTCCTCCATTGGAGCAAAGCTGGCCTtCAGCCT

CTCATTCTCCAGCTTGAATGCGGAATACTCAGCAGTCTGACGGTGTAGTCTTTCCACCAG

GCTATCCCGGTCTTCCCGc

>contig06275

CTGATAGGCACCTCAGCTgCTAAAGATGCAGCTCACACACTGGAGGCTAACAGGAGTTAC

GGCtcGTGTGCCGGtggccaacaaaagggaCACGCGCTCCATAGAGGAAGCCATGAACGA

AATCcGAGCGAAGAAGCGGCAGAAGCGCGAAGACGACACGGGGGCACAGAGCAGCAGTTT

CTGAGTTCCCGCTGTTGCTGTTGTGTGTTACTTTGTGTGCGCAGTtCGATTCTTCTGTCG

ATGCAGTTTTCCATACTTTATGACTTTAAAGTATAGCTTATTGATTGGTCAGGCTTTACG

TCTGTATGAACATGACTGTGATCTTGCCCAGTGGACGATATTTAGGTTTGCGGATTATTG

GCGACTCTTTAACATCTTCTTGTCACGTTCTGTTAGTCATCGGATGCCACGTGCCATTTT

AAaCCGTAACAGTATGAGTCAGTtGTTTGGAAAGTGTCTTGTCATTCaGAAAAAAaCATG

ACGATAGATCATTTTTAAaTAAAATCTTTTGTTGCTTAATTATAATTTTTGATGCAGCTG

CATATTTAAAAAaTGTTAGGAAAAgTTTTATTATTTAAAATCACTGCTAATTTTGTACCT

TGGCTGtGTTTTAGCAAGTTGCAGCTGCAGATTTCCATCGATAaTGTTTAAAAATCCCCA

GTGCATGTAGAAACAAACTTTACCTCTTTACCAGCTTcTTGTCAATTAGTTTTTAATTAA

GGGTCAaTTTTTTCACTTTGAAAGGTTAAACCTCGCTAAATGTGTCCATCACAGGAGTAA

GCCAGTTTtAAAaCCcACTTtATCTGAATTTTtAAATGTGATTTTtGCTTTGTTTCTACA

CATTGGCACTGACTTGAGTGTGAtAGTtttAATTTTTTTTTTTtCcATTTTAAGTCATGC

AACAGGTGTCGTACTACTAAAATGGAGTAAAGATACAAATCTCCTTTTAGCATTAAGATT

TATTTAATCATCATCATCAGACTTTGAACCAAGGAGGAATAGCGTGGCGTCATTATGTCT

TTTTAAACCTTGTTTTGTTTCCATGTGTGTTAATAAATGCTGCTTACAACTTTGtGTtaC

CTTTACTTTTGGCACGCTTCTtATTTTGTTAGCaTTTtAaTCCACCAACATGCTACATTA

AAaaCaTGCTGTATAAAAt

>contig06905

TGACGACGATGAAGACGAGGACAAAGAGAACACACAAGATAACGAAGAGGAGGATGTTGG

GAATTTGCAGTTGGCTTGGGAGATGCTTGAGGTGGCCAAAGTCATTTTtAAAAGAAAGGA

AAGCAAaGACGACCAGCTGATGGCAGCACAGGCTTATCTGAAaCTGGGtGAAGTCAGTGc

aGAAaCAGGTAACTATCCTCAGGCGCTAGAAGACTTCCAGGAGTGTCTGATTATTCAGCT

GAAGCTCTTGCCGCCTCACTGTCGCCTGCTGGCCGAGACGCACTACCACGTTGCTACGAC

GCTGGTCTTCATGGACCAGTACGACCAGGCCATAAAGCACTACAACAGCTCCGTAAAGGT

CATCGAGACGCGTCTAGCCATGCTCCAGGAGGTCATTGACGCAGCGGCTGGAGATGACGG

TGCTGCAGAGGAGAAAAGTGAGCTGGAAGAACTCAGACAGCTTCTTCCTGAAATCAGGGA

AAAAGTGGAAGACACCAAGGAGAGCCAGAGGACGGCCAGTGCTGCCTCCCAGGCAATCCA

GCAGACACTGGGAGCTCCCTCTACTTCATCAACATtCCCCTGTGAAAaTGGCGGCCCGTC

ATCCTCTACTGCTTTTGCATCGACCAGTCAGATTCCAGTGAAAACCTCTGAAAGTGCTTT

GtCTTCCAAAGCCGTGTCGGATATCTCTCACCTTGTTAGGAAAAaG

>contig07134

TtAAtCTTCTGTCTCCCAGGACACCATCAACAACGTGAAGTCTCGCCTGGGGAAAAGCGC

GCAGATCCAGACGCTGCTGCGGGCGTTCGAGGCTCGCGACCGGAACATCCAGGAGAGCAA

CTTCGATAGGGTGAACTTTTGGTCGGTTTTCAACCTCGTCGTGATGATGGTGGTGTCGGC

CGTCCAGGTCTATTTAGTCCGCTCGTTGTTCGAGGACAAGAGGAAAATTCGCACATAACA

TCGCCTAGAACTGGACGAACAGAAATCATGAAACTAACACACGTTAACACGGTTACATTA

GAGATGCTTTTTTTtGTTTGTTTGTTTTTCTTTTCCATTATGTCTTTTAAATGTTTGCAG

AAACCAACAGAGGTTCAGGACTAATTCGCCACTGTTGTTGCTTCAAGTTTTCTGTCCACT

GGCGTTTGGGAATGAATCACAAGTGTCGACTAGCCATACCTCCACTCAACAACCTTTTTA

CAAGAAAAATGTTCTTCTCTCTCTTTATAGCTTTATACTGTTTTAAGGTCCTTCTTAGCT

GCTGTAGTGGTTTACACAACACGTCATAACAAGTTTACGATATGCAACTACTTTTCT

>contig07643

tCAATCTTTAACTTTCACTGATGCAAACAACATAGCAACATCACGATTTTGCACACTAAC

ATAGCTCCTCCTATATGACAAGTTTATTGAgaaactaatgtctttatgttaaaatgacct

gaggcagaatatttttagcctgttaccctccatggtaaaTCTGGGTTGTTTAAAaaaGCT

ATTGAAGAACAAAAGGAATGGTGAGATCAGGTTAAGTGCTTTATAGGTTGCTGTTGCAGT

CACGGCACAGGATCTTGTCGCCATCAGGGAAAAAGCCAGCGCCCACAAGTGAGATGGAGC

ACTGGGAGCAGGTGAAGCACGGCTGATGCCACTGACGATCTTCAAATGAGATGTACTTGC

CTCCTCCAAATCCTGTAATTGGTTTGCTGCAGGCCTC

>contig07774

aaTTAGTACTTCACCTGAGGCGGGAGCTGAGAAGATTTTTCTGATGTATTTATATACACT

GTGTATTTCTGTAAGCCGTTACATGTCTAGGAAATGCCCACTAACTATAATTTCGATTTT

TAATTTGTGAGGCTGTATAAATTGTTTAGAGTTCATTTAAAATAATTAAAaTTGTGCTTT

AAACAAACTTTAAAATAATAGAGGAAACAACACATAGTTGCTTTTGGCAAATTTCATGCC

TCAACGACTGGGTAGATTTTAAAATCTTTGTGCGCTGGAAGGAAAATCAAAAGACCCTGA

CCTTGTCGGAGCGGCCGTTGTTGAGGCTGAGGTTGCTGACGGAGGCCATCTTGGCGTCCA

GGGCCTGCTGCTCCCCCTTCAGCTGCTCCTTCATCTGCCGGGCCTCGGCGAAGGCCTTGC

TCaCCGCCTCGTGGTCGTTCAGGTAGGCTGTCGACGACAACGAAGACAACAAGTCTGCTG

AGACGCAGACAGGAGGGACAATCTGTTAGCCATCCTGTTCAGCTTCTTTATTTttCATAA

GCACAAATGCAGATTgagtattaacagacaaaaaaataacaaaaaaaaaCAGCCTTTttA

AAATTATTATTATTGGaTAAaCACTCCAAAGTGTTTACTTTGAAGGTTCTCATCTGGAAC

AAATCTTCTGAACCATAAGGTAAAGGCATTAACATACATTTGATGAGGGGAGGTTGCCAG

GGTGTGTGTGGGCGTGTGTGTGTGTGtgTTTTTttATATATTTAACTGAAAACACTCAAA

GCAACTGACCACTTTGCAGCCGAGTGGAGCATGTGAGTCAGAGTTTGCCCCCAGCAATCT

CATCCCCTTCAACAGAACGCTGTAAGCAGCGTTACAGTCAGCAGAGGCACCTCTTATTAT

TCTACATGTGTGAGAATGTGAGGCGGCGCTAACTCCGCTCCCCATCGGTCCGGTTAGTTC

TTAACAAAGAGCCGCTGCTGCTTTATGAGGCCTTTATCTGCGCTAATCCGCGCTGCTAAT

GCCATCTCGGACCAGATTATCGACTTCAGATTGGTAATGACAGAGCCGTGAACTCTTTCA

GAaGTCATAACCGGAAAATCTGCACGGGAGTGGAGCCTGACaa

>contig08354

aaaaGGACAATTTTGTTTAGtGATAACATgATTTATGTTATTATTTCTGTTGTTCTTGTT

TATTTATTTGGGCATTTATAATTAAATGTTCATTAGAGTTTAAAGTTTATTCCTCTTTGA

GAAAGTGTCCTTGCATTATTGTTCCATCATATCACTTGAAAATGGTCACAAAGCAACAAT

ATTATCTTTTATCGGGATAACCTCTGGGACAATTTACTGTCCAGAAAAATTAGTTGTTAT

TGTGGAAGGCCTATTCACAATTATCACTAAATGTCACTTTAAAGACGTCTCTTTTTTTCA

GTCCACCCTTCATGAAATGTTACCACATTTTGTAGTAAAAAAaTAAACATTCTTCCCCAG

GTTTCCCTACATTATAGCCATGATATAGGATAGATGACGgACaaaa

>contig10040

aTCCTCACTTGGCATTGATCTTGGATAACTGCTGGGCTACTCTCCGAAAGAaaGGaCCTC

TCTCCCCAGCTGGGACATTATTGTAGCCAGTTGTGCTACTCCGGCGATGGCTATGCAACA

GTCTTCAATACCGTCTCAGTGGATGACAGAGTTTCTGTCCCTTCCCGCTACAAACGCTTT

TCAATGAAGATGTTTGCcTtCATTCAGGAAAAGCAGGTTCTGAAGCATGAGGTTTATGTC

CACTGTGACTTGGTAATTTGTGACACAACCAGCCCTGCAGAGGGTATCTGTCAAGGCCAA

TGTGCTAATCTTGCTGTGAAGGGTACTGCAAAACAAATGAGAAGGGGACAAAGAACACAA

CCTCAACCCACCAAAAGCAGATTTCCTCTGGACCTATTACAATGAACAGTTTTCAAt

>contig09487

CGTGTTTTGAAGCGGAGTGGTCTGAGCtGCTGCTGAACGGAGACGTGGACGCGAAGCAGA

TCGGCCTGATCCCGAAAGTGTACACCGAGAACGAGCATTTGGCCCGAGAGCTGGAAAGTG

CTCGGCAGGACAAGGAGGAGTACACGCAGGCGGCCGCTGTCGCAGCCGAGGCCCTGCAGA

GGgCCAAGAGAGCCAACGATGCCcACCGGCTGGAGCATCAACGCTTGGtCAGAGAGGTAA

ACAGACTCATTGTGGACATGAATAAGCTGAAACTGCAGTGTGAGAGCTACCAGCCTGAAG

TAAAGAGGATGAGTGACAAATACCAGGGGCTGTCAAAGCAGGTCCTGCAGGTGGCGCTGG

AGAGGGACAAAGCGCTACTGCAGGGGGACAATCAGGCTGCCCGACTGGATGCCTCCTCAT

CTCAGACCAATGGAAACAGTGGTTCATTGAAATAAATCTCAACTTCATAACAAA

>contig15766

gTAGAGAGAACCTCAAGAAGTCCGAGCTGGAAGGCTTTTACCCCCCAGCTCCTTTGGAAA

TTCGGGATCAGGTCAAGTCATATCAGGAAAGAGGTGCATGAAATGCTGATGAAGCTGCTT

TGTCCTCATCCCTGAACTTGGCTTCCCACTGGgaGGAAAaCcAGGACACTCTTGATTACA

CAGCTGATCGCTGAGAGGTCAGACTTTCACTGACCCAGATCCCTCAGGAGCAGGACATGG

ATGTTCTCCGGCGATCTTCTATGTTCGCCTCGGAGGTCCTCGATGtCTTTGACCGATCGT

TGACCGAGAAGGAGCTGGTGTCTCAGTCCAAAGCACTTTGCAGAGACTATATTCTATCCA

GGCTCAACCAGAACGGGTTGGGTTGgTCCAAGACTGAAATTAACTTCTCTGCCCcAAATG

CAGCGCTCGCCGAGGTGTCTCTGGTGCTTCTTTGTCTTGGCGATGAGTTGGAGTGCATAC

AGCCCAGTCTGTACAGGAACGTGGCACGGCAGCTTAACATTTCAGTTGCCATGGAGAACG

TGGTTTCAGATGCCTTTCTCGGCGTGGCAACGGAGATCTTCGCAGCAGGTAtAACATGGG

GTAAAGTGGTTGCCATGTATGCAGTAGCTGGAGCCCTGGCAGTGGACTGCGTCAGACAGG

GACATCCCACCACAGTGCACATCATAGTGGACAGTCTTGGACAGTTTGTCCGCAAATTTC

TCGTTCCCTGGCTGAAGAGACGCGGAGGATGGGCAGAGATTATGAAATGCGTGGTGAAGA

TGGACTGCACt

>contig11497

TtgCGGGTGAGAAGATCAAGTGCttaTTTTtATtGAAACGACCATCAGAAACACAATTTT

GaTGTTGCCAAAaaGAAGAGGTTTTCCACAGGAAATACTGACTGCaGTACTAATGatgTG

ATTtTttCcAAAAGTGGGTCCTGAAaTTTAaCTtaTTtCCCATCGGAAaGGgcAGAAGAT

GACAAGGGCTGGAAgcTTGAGCTTAGGCCTTGGCGGCGTGGCTCCTGAGGTATTcGACCT

TGGCCTTGAGGTCGTATTTGCCGATGAGCTCGTCGCACtGGGgCCAGCATtgAACGCATT

CGGTaGAGATGCAAGAAaGGCAgTCAGCACCGCCGCTGAGGCAGTCCCAGGCGCAGACGA

GGCAGTCAGTGGCGCAGAGAAGGCATGTGGCCCAATCGTCGCGAACTTCCTGGTTGGaTG

GGATCTtGGCAGCCATGGCAACAGCCAAAAGAGCGAAAAAGATGACGCACTTCATGTTTA

GTTTTTtGGgTTTtGTGAATAAGCTTTGAATAAGCCTTGCAAATTTGAAGATTTCTTAAA

ATTTtGCAACTGCTTTGAACACTTGAACTCTCCCCGC

>contig13920

ttCtaGAACTCTTtACAGTGGAATTGGAAtAtATATTTTTttCCCAAAATAAAACAAGCA

AAAATAaTAAAaCAATAAaTAAAAaGGAAGTAAAaGTGGCAAACAAACAAAACCATAAAT

AAGACGAACTGTCTCTGTAAACAACATTCCCTTTCAAAAAaaTGTTAATCGTCAGAATGG

AACTTAAAAAAaaaaaTAATAATTTCCCCCAAAaTAAGAATTTCTAGAGACAAACCCCCC

AAcTAGTTCATTTGTAAAAACACACAACTGTATCTGATATCCTTTTAGAATAATTCATGT

AAAAAAAAACaTTCTTAAACTGGTCAGGCACTGTAAAATATAGCAAATTCCTTGATATTA

AAACGCATAGTGGTCCATTTTCAACAGACCTGCGTCTCTGGAGGCTTTTTCTGCTCGAGC

CTGCAGGACCTGCTGGCTGGGGGGTTCTTCTCGTCGGTGCTGCTGGGCGGaCAGGGGAAC

CAGGGGAATGTCTGGGAGTTTCTCTCTCCACTCTGCAccGTCCTCCTCGATCAGCATCCG

AGTGATTCTGGTGAGAGaGAAAAAaaCaGAAaCAGAGCGGGTTTGAATAGAGCT

>contig14446

TCCCCTCCGACTCCCTCAGTCCAGGCATCCGAAGAAAAGTCCCGAAAAAAGCAAGTTATT

CGCTTTAGAGTTAAGCTTTCCAAGGATTTGCAGGGACGGGGTTTGCAGTAGTGAAACCCA

CTGGTAAGCAAGAGCTCCTCTGCTTCTGTTTGCGTCTGCGAGTTTTAGCACTCAGTCAGT

ATGTGAACACCAGGTTGgAGATGGTGGACTCCAGCCAGTCTCCCGATATCATCTCGCTGA

CTTCGGGCGTACAGTAGTCGGGGAACTCGAAGTGGGaaCCCCCGCAGACGGACTCGCCGT

TCACGTCCCAGTCCCTGTCCAGGACCGAGGAGCCGAAGCTCCCCAGCGACATGCTGTCGA

AGCTCGGACTCGGGTTGACGTCCAGCAAGTCGTCCTCGAACTCCTCGTCCTCCGAGGAAG

ACGAGGAGGAAGAGACGGAGG

>contig14696

TTTTGGCGATTTGGATGCTTTAATTGTTTTTGTAAGCAAGTCAGAAAAAGGTCAAAATAC

AAGCACATCAAAAAACAAAaaCAACAAAAAaaCGTGGCAAATCAAAAAACAACAGAAGCC

ACCAGTCACAAAaaGTGATGCATACAAGGTGTATACACAGGATATTTGCACTGATCCATT

CAAAAAaTAaGCCCTGTTTAACTTTtGGCAGACATTCATTTTCACATTCACAGATCATCA

TCTAAAAaCTAGCACAGTACCAGAAATTTCACAAGTACATGCAGTGAGTCGCAAATTCTG

GGTCATACATATCCGTACTGATGAGGCTGCCGAAGAAGCAGGGAGGGAGTGTTAAAGTTC

ACAAATATTTCACATAACACACCCTCGAAACTGTTCATTTTCTAAAGCTAACTCATGTTA

GGGGGGAGGTGGAGGAAGGGATATGATTTTGTTGGGGTGGGATTAAAAaGGTAAGCCTGC

AAACCAGCTTATGCCAGCTTGAGCCACACATCTTTCACTACCTCTTCTTGACTCTTCTCC

CTCTCATCATCCAGGTCAGGACAGGGGCGTCACTGCTCCGGCACAGATGTGTCGTCTCTC

TCTACTGCCCCCGAtCTTCCTGTCTCTCCTCCCCTTCGGTTTTGTCcTCTGGCTTCATCT

TCTTACGAGTCATGTGGCCGCGGAAGCCAGCCTGGATCTTGGCAGCAGCTCTGTTGGCCT

CGGGGTCgTCCAGGGGGATGTCCAAGATGTCCTCCTCCTCCTGGGGTCGGCTGCACTCCT

CCTGATTGTGACAGTCCATTTCTTTTGCTCTGGGACGACTTCAGTCTTCAAGTGAGCTGA

TCTAGTTTAACTGGAGTGCCTAAATAAATTCTGACTTGGGCCAAGGAGGACGCTCTTTAA

ACGGGTTTCTGATACAATACAGATGCCTGATTGGCTCTCACTAACAGTCGCTGTATGTTc

>contig20615

gAAGGGAAaCAGACAAGGTTCTAAGTCTAGTTAGtCACtAAAGATGATAGAACTCAGAAC

TAAGgaTGGgATGGcGAGtttttGGGAACTGTGAGGTCACTCATAGGCATtACTTACTGT

AATCTGTGACGGTCTTTGGATTGGGGTAGATGTGGGCTTAATCATGATGCTGCTCGTGAT

TTTACTGCTGCCTTTGGCAgCGGGAgTGCTGCAGTTTGCAaTAATCTGAGtCTTTTGCTC

CTGGAGTCCAGGGACTTGTGAAGGGGcCTGACgg

>contig18544

gggTGCTTTCTGGCTTGCAGTGCTCTTGGCAGACATGAGGCAACGATTGCTCCCGTCCGT

CACCAGCCTTCTCCTTGTGGCCCTGCTGTTTCCAGGATCGTCTCAAGCCAGACATGTGAA

CCACTCAGCCACTGAGGCTCTCGGAGAACTCAGGGAAAGAGCCCCTGGGCAAGGCACAaC

gggtttcagctGCTACGCCACGCAGTGAAACGGGACCTCTTACCACCGCGCACCCCACCT

TACCAAGTGCAcATCTCTCACCAGgAGGCTCGAGGACCCTCATTTAAGATCTGCGTGGGC

TTTttAGGGCCTAGATGGGCCAGGGGAtGTTCCACGGGAATTAGAAATACCATCTGCCGT

ATGCAGCAAGGGATCTGCAGACTTTTTTTCTGCCATTCTGGTGAGAAAAAGCGTGACATT

TGCTCTGATCCCTGGAATAGGTGTTGCGTATCAAATACAGATGAaGAAGGAAAAGAGAAA

CCAGAGATGGATGGCAGATCTGGGATCTAAAATa

>contig20031

TCTGACTTTCAtcAAAGTTGTCGGTGAGCAGCTGCAGGCAGCGGTAGAGAAGATGGGGAA

CCGAGTGGGCCGTGAGGACTACGAGTGGGTCTACACGGACCAGCCGCACGCGGACAGAAG

AAAAGAAATTCTGGCTAAATATCCAGAAATCAAGTCCTTGATGGGTCCAGACCAGAGGCT

GAAGTGGATTGTGTGCATGATGGTGGGgATCCAATTCTTAGCGTTCTACCTGGTCAAAGA

CTTggACTGGAAGttggg

>contig20152

TTTTAAACTGTATTTTAATCTGAGATTTCCAAACAATGAAAAAaaaaGACCTttACAGTC

CTTCTAACAGTGATTCTTTTACAACATAAATaCTGTCCTGAAAAaCAAATTCCCcAAAAA

AaGAAAACATTTCTTCTCAGCAGTCTCTCTTTGTGAATATTGAAAaaTTTTAATCGGTTC

TCCTCAGTTCTCAaCaaGACAACATCAAAATAACAACAACCATTCCATCAGTGAGGCCCA

GCCGCCTCACtatgtagatagtctctaatggagtccttttgtttccttcatggttctaat

tcttttAAAGTTCTAAGGTGTTAACAGTGCTCACTTTtAGATCCAGAAGGAGGGTAAGGA

TGTGGAAAAAGTCTTGAGTGAgCAGGGAGAGGTGGTGATGATgAGGGGTGAGGGGCTAGT

GCTCTGACCATCAGAAGAGGCTGGTCAGTGTTGTCTGTGATGGGCTCCGGCACTCGTGGA

CGTCCATACCGCCCGGCACCGACGTGAGTACAGAAGTGACAGTAGGCATGGTGGGGTTGG

TCAAGTCTGTGAGGGTGGTGGGGgTGCTGGAGGGGCTCATGgAGGCGTTGGaGATCTCCG

AGGCCGGGCCTGAGGGCGTCCCACCCTGCAGCGTGGaa

>contig20381

cTGAGAAGGCAGATCATTCCCCATCAGCTtCCAAaTTtGCATCGTGTCAtCAtCtGCACC

ATGAAGTCGTTGGCTGTGTTCCTCCTTCTTTtCTCCATCATGGAGAAATACTCACCGgCA

CATGGTGACGAACAAGTTCATTTGTTCAGGAGAACTGTTGAGTGTCCCTCTGACTGGACT

CcAgtCAACaTcGgCTGTTTCAGATTTGTTGCCgATGCCAAGACTTGGgCTGgAGCTGAG

AAAAACTgTATGTCCTTGGGGGgAAACCTTGCATCAGTTCACAGC

>contig22779

aGACAGAGTTTTAACACTTTTATGATGtttCCTCTTTAAATTTtAGGCACTCAGCAATAA

AAACAAACACATTAGGAAAAaCAGGCACTAAGACAACACTTGACACAAATTaGAAAAAAa

TaGTGTTCgTTGAGTTTTTGGGACGGTTTAGCGGAAGGAACAAAaGCCAAaGAGCCTCAT

TTCTTGAAGAAGGCGCTGCGAGTGATGCTGTACAGCAGGTGGTAAGGCTTTCGTCTGGGG

GgTTCGgCCAGAGAAAaCACCTGCTGCTGAGGCACGCTGGTGGGAATGGTGATGTGACGC

TGCTGAAGGGGGGGCAGGTTTTGGTGTGGCggg

>contig22887

aCCGCTGACAGCTGAAGGCGAAGGCTGCCTGTCTCCGGCTCAAGGCTGTGAGGAAGACTC

AGTTGGTTAAGGTGCTGGAAGCACGGAAGTAGGAGAGGAACTTGAAACAGAGGCTGACCA

CAAAGTACCAGATGTTTCTGGCCATCTGGGAGCGGGCGATGAACACCCGCCAGATGAAGA

CcACCAGCAGGGTGTTGAAGGTGTACCGGATGTTTCTCAAcTTTCGGAGATGCtaGCCTG

GCAGCAGGAATGTCGGACATGTCTTCATTCAGGACGTACTTCTTGGtcgaCCGATACAGT

AATTCTCAATGTATTCGGGCCAGTTCAGCTGGCGCACGTCCCCGC

>contig30745

TCAGACAGGCCAAAATGGATTACTTCTTTGATGACCTTCCATCCAGCTCCCTATCTACTC

CTAAGAAAGGACATGAAGACTCACTGATGGTGGAAgATACAGATTGTGATTtGTCAAGAA

ACAAGTCATCAAGTGTGAGCATGACTGAGCTTCAAGAGCACCGTAGTAGCTCATACATTG

ATGACATCAGCAAAAGAGCACAAGAAATGGTGGAAAAAATAAaCCATGAACGAACCAGAG

ACCAGGAAATGATGGACAGCTTTCAGAAgCAGCTAACAGAGAAGGTGACAGAGATGTGTC

AGCTGATGAAAGAGGAAaTGTTCACCATCTATGAGCTTAACAGCAATGAAATCCAGGTGA

AGCTGCAGGAGCTGTCAGAGGTGCTGGAAAACTGCTCTAAGCTGGAGCACGAGCTTCtGG

AGGCCAGTCAGGCCTTGGCATGCCTCAAAGATGGTCTCGACATTAATCAGAGAGCAGAGC

CCTAACAAATTAATTCTATTCTCAACTGGGGTTATTAAATTGAAGACa

>contig24990

gTGCGCCAACTGGCTTGGGTTgAGACGGCATCTGGGTGTCCATCCGACTGGATTCAACCC

TGCGCTTGTTtCACGCCCACACCTACCAGCACCTGCAAGACGTGGACATTGAGCCTTACG

TCAGCAAAATGCTGGGTACCGGTAAACTGGGCTTCTCTTTTGTGAGAATCACAGCTCTGG

TGGTTTCCTGCACCCGACTgTGggTGGGAACAGGAAATGgAGTCATCATCTCCATCCCAT

TATCTGAAGCTAATAAGACCACAGGTATAGTGCCAAATCGCCCTGGCAGTGCTGTGCGGG

TTTACAGTGATGACTGCTCAGAAGGCGCCGTGCCGTGCAGCTTCGTGCCGTACTGCTCCA

TGGCCCACGCCCAGCTCTGCTTCCACGGACATCGGGATGCGGTGAAGTTCTTTGTCACAG

TTCCAGGTCAAGCGATGCCACCTCCAGGCAGTGCAGATTCAGGTTCTGATGATCCTCCAT

CTGAATCCTCAGACACGGCAAATTCGGAGCCCAAAaCATTCCTGGTCATGAGCGGAGGAG

AAGGTTACATTGATTTTAGAATCGGTGATGAAGG

>contig26089

ggCCAAGATCTTCCCCCATAAGAAGCGAGACGCCCTGGAGTCCATGAGCTCAGTGTTCAA

GCCTGCAGCCGGATTGCCGGGAGCTCTCGGTTCTtCAGGGACCAAGTCCGCCTTCTCCCC

TCTCCACGTCCACCCGGCTCCCGGTGGGGACAGCCTCTATGGGCTTAGTCCTCACCTTGG

TGTGAGCCCCCTCGTCTGGCCTACTCCTCTGCCAGCGGTGGTCTGGGTGGGTTCATGTCC

CCGTATGTGAc

>contig27348

aGACGATGCCGGAGACCCCAACTGGGGACTCAGACCCGCAACCTGCTCCTAAAAAAaTGA

AAACATCTGAGTCCTCGACCATACTAGTGGTTCGCTACAGGAGGAACTTTAAAAGAACAT

CTCCAGAGGAACTgcTGAATGACCACGCCcGAGaGAACAGAATCAACCCcctCcAAATGG

AGGAGGAGGAATTCATGgAAATAAtGgTtGAAATACCTGCAAAGTAGCAAGAAGCTACAT

ctcTCAAaCcTTGGgCAATGAAAATAAAGTTTGAGAAG

>contig27967

tttGttGAATGCGGCATCCCACAAATGGGCCCTTTATTTCTGAACTGCCACCACTGCACG

GATAAGGCCACTTGGGTTGCAGGCACACTGTGGAGTGTCAGGGGACAGAGAGGAGAGGGG

CTGCACACCAaGACTGCCCTGTCCTCACGGAGAGGAAAAGCGGCCGCAGAaGAGAATCCC

GTTGGTCTTGCTGTGCTGGATGAAGAAAAGGAAGGGGTGGTCGGCGCAGAAGCGGGGGAC

GAATCTGGCACACCGCATCAtCATGATGGCAgctgtggcggctgcagcctccgtgccttc

ctcattgacctccacaaaagacttgtgcacgaccttggacagagacaggtctgtctggga

cattccagagaagtctgccttgcccagctcgaaggcATCAGTCATGCCCAGGTTGCGCAG

GACACTCTCCATGTCGTAGCTTTCCTCTAGTTTAAAc

>contig30170

GACGCGGGgATCAGCCGCTCTATTTGCTGCACTATGATTTGGAGGtGAGTtAtGTTTTTT

CGTAGTTAACATTTTtAACtGAatAAACatAACCACATATGATAAACATATATAAAAaTG

ACCTTTAtATATAGTAATAAACATTTCCATATATTACCTCCGATGTCGACCGGACTCTCA

GTTGCCATGTCAACTGTTTGGGATTCCCTCCATTCTTACGTCACCACACTTTCTGATTGG

CTACCTGTCACATTCAACGGGCTGCGATAAAAAGGCCAAGACGATGTTGAATATGATTTT

AGTTCAGACATATTCAACACTCCACCATGTAACACATCACACACTGCaGGACGTTGTAAG

ATTGTGGTAAAGGGAAAATTGGGGCAAAAAa

>contig30184

tttttttGCACCTAGCAGCAAAAaTGTATGCTAAATTACTAGGAACACACCGGGAGAAAG

ATTtATTtATTttATTTTTTTtGCTTACcTGTTTTAGATTTAAGCGCAAGAAAAaGTGCA

AACATATATCAACAATAACTGAATATaGaGCACAAACCTGGGAAGCCAAGTCTTCAGCTA

GTACATTTATATTTTTAGGATATGATCTGATAGCAAAGAAATGATTTCTCTCTTAGCATA

CAAACACTTTTAGCATTCCGCCTTTATCCTCCAAGAAACCAAAATGCTGCTTCATGAGAA

CAGGTTATAGATCATTCTCTCTTTGAAAACACTGGCCGTCTttAGCCTGGTTTAATCAAT

GGTTGGACAAAACGAAATGTGACCTTTAAATGTCTGTAAAATAAATTTAAAAAGAAGAAG

TTCAGGCTGGATGAACTACCTGCGGAAGGCCAGATTTCTATCATTCCAGAAATGTGGCCG

CACAAAATTATTCCTCCAGCCGTAAATGAACAGGCCTGCTCTGAGAACCGAGTCTCATCT

CTCTCCAGCATATTATCTTCCAGGAAAGTATGTTTTCCTTTGGTTTCCTCATTCCGACCG

GATGGGAAAACTATCATAGTTCTCCTCCGCTGTCCCCTCCACTTCCTACCGTTCACCTCA

CATTACCTCCCCATGTTTCCATTTTCCTCCGTCCCCCAATTCATCCTCTCCCCTCCTGTC

TGACTGCACATGAAAAAGGaGCTACAGGAGCCGCGACGGGCGGGGCGGGTAAAA

>contig30427

TTTTAAGAGTCATCTTTTTACTTTTATTGGTGCGTCTTTGCGTTCAATAGATAATCACAC

CTCAGAAAACCACAAGAACAAAACTAAACTGGGCAAAACATATTACATATTTATGAACAA

TGCACAAAACTTCACATCTGCAAGGAATGAAAATCATTGTAAAAATCAACATTTTTGATT

TTACAAAACCAAGAAATGAGCTGAATCTTAGCGAGAGGCCAGAAACGCAGCCAGCGACGG

CTACTTCGtCTTTTTGCTGGAGCTGCTGTCACTGCTGCTCGGAGAGCTGGGACTTTTAGG

AGAAGTGATAGTAGGATAGGCAGCACTCAGGTCCAGGTTCAGGTTTACCGTTTTTACCCA

AGGCAAAGACTTTGAAT

>contig30428

ATCATCTATAttAGACGCGGACAGCATGATGTCATGGGAGATGAGAAAGTTAGAGCTGGA

GAAAGCCATGAAAGAGCGGGAGGAAGAGATTCACGTTTACTCCGAAATGCTCAGACATCA

GCTCAAGTTTACCGAGCTGGACAGGCAGAAACTGAGCATGGAATTAAATGAAGAaTTATC

TAAAGTCGAAATGGTGAAGAACCGCTTTGAGATCATGgAGAGTTCGCTGGCgggctctga

gggcgagaagtcccagatttactacatcactaaGGCCACGCTAGACAAAGAGGAACtCAG

aCGGAaGTCAGAAGAtATGAAGGAGCACATCCAGAAGATGGAGCTCGAAACCAAGGCTCT

GGAGAACACCAtCCACTtGTTTtCGGGTCGCTGCTCTGATTACAGCAACGGCCTCAGCGA

AATGAAGAAATTCAGCGAAGAGTACCAGGAAAAGATGCAGCTGGAGGAACGGCTGAAGAC

CGTCGAGGAAACGCTGAAGTTGAAGAAGCGGCAAATTCAAGAGCTCCGTCAGGAAATTCA

GGACATGAACAGCACCTGGGAGAGCCTCATGCTAGAAGAGAAGGTGGAGAAGGAAAAGAC

ACGTCACGTACAGGGTCTTATTGCTAATCTGAACAAaGAAGCAGCTTCTGCgCAGGAGAG

AATTAGCAGAGTCATGAATCAGTTCACCAAACTGACCAGAGAGGTTCGCtCAGCACAAAG

CACCAGAAGTGAAACTCTTGAGGAGCAAGACATCAaaCTGAAAGAGTTAAAGCAGTTCAA

CAAATCCATCAACAATATGTTGAGTAAGGCCATGGAGGAGGATCCTGATCTCAGACCTGT

TCTGGAGGAACGCTTTCAGAAGACCAATCTGCAGTTTCCACCTCCGACcTCCACACTCAG

CAGCCAGggTAGTTTCAAGATGAGCTCTGCTCGAAGCTCCCTGTCTTCCAGGTCTTCTGC

GTCGTCAGCTGGCAGCAGCC

>contig30599

GCGGGgtAgAtCAAAACAAACAATTAACCTGCAAGGACACTTTGGACTTCTCTCACAGgT

GAaTGTGCTAGATCAAACATGGAGAACTTTCTGGTGAAAGAAAACAACAAGACCAGCCCA

ATGATGTATTTACCAGACAACGCTAGTGGCGCCCACCTGAACGCAGTTCACGATGAGAGT

CTGAGCAGAGCCGGTgACACTAAAAGTGACCAGCTCCCCCGTCCTGAAGAGAACAGATCA

CTTTTTGAGAGCCTTCCCGCTTTGCCTAGGAAGCTCGCCTCCAAaCGTGGGAGGAGGACG

TACATCTGCGACCAGTGTGGGAAGACGTTCCGGAGGAAAAGGCGTCTGACGGCCCATCTG

CGGAGTCACGGCGTTGAGAGGCCGTACAGCTGTGTCCAGTGCGGGAAGAGTTTTATACAG

AAGGGGAATCTGACGATCCATCAGCGCGTCCACACCAGAGAGAGTTTACCTCAGGCCTGA

ACTGGAGGGAAGTTTATCAGAACCTTGTCATCAACGTACATCGACCACATGGTGTGAAAT

GTACATTTGAaCTTAACAGCTAAAATAAAAGTAATTCGGAGAGCAAAGTAAa

>contig30607

tttGCATTAAAACTTTTGCGATAAATTTACAGTTTTCATCCAAACTTGAACGTTTTGGAC

aGgAAAAaCCCTGTTGCGCATTAAaCAAAAATCAAAATTTCAACCCTAAATCTAATTCAC

AACATTATCGCTCGCTGTGTCCTCTTCAAAGAGTTTATTTATCATAACGCGAGCTGCTGT

TTTCATATTTTACAGCAAGTCTTTTCCTGTCATTTTCTGACTCTATTCTGACATCTACAC

TCCAAACTATTTCCTAACGTGTCATAAATTAAATTCTGCACAGTTCTGAGAGGCAAGCGT

AGCGGGGAAAAGTTGAGTTTCGTGCTTTACCTTTGCGCTGCAACGTTCGCATCCACAACT

CCAACATTACGCACCCACGACCGGACTGGATCCATCTCCTCTCCAAGAGTTCTCTCTTTT

AATCCGCTCACGTCTCGTATTAGCTGCTCCTGGATCCCAAACATTTAAAGCCCTGTCCTG

GTCGTTTGGATGACTTTTTGAAATTCTGTTATGATTTGGTTCGGACCGACAAGATGGCCT

GGACAAGTTGAAGCACTTCAGTAACGAACAGAGGGTCCACTTCGGGGCGCCGCGCTCCCT

CAAAAGCGCCGCAAAAGAAGCTTCCAGTGAGGTTTGGGGGAGAAAAACAATAAATAAATG

TCTGGTGCTGGTGTTATGACTCTTAACGGGGCTCAGTAGAAATCCGCACGGACTTTCTTT

CATTCAAGGCTCAAAAGATGAATGTAGATATATGTCTCCACaGaTTACAATCATTGTCCG

AGCGCACATCTCGAGCAGGAGTGGGAGCGCAGCGGATCATCTGCTCCAACAGAGAGGATC

AGACGGAGATGGAGATGtaCTTTAAAa

>contig30647

TGggtAAGGTTTTTttATtGATGAAAaTGTAAACTCCAAtGtAATTtCAtCAGAGCACAT

TTCTATCTTTtCTCTCATGTTCTTCATCTCCCTCTCCTCTTGATCGTGCTTGCTGCACTT

AAAGTGACACTCACTTGGAAaCGTGGGCCTCCTCTGTTGCTGTTTCCTTTtCAAGACCAA

CAGTCTCTGTACTCTTGAAAGCATGGTTATTGTAGAACCTGTCTTCCAGCTTGGCagCCC

ACTCAGAAGGGTCCATCCCACTTTCTTCTCTTTCTTTCAGAAGTTTGTCAAAATACTGCG

CTGCATATGCAGGGATGTTGTCGGGCTGGTCCCGGAGAaTCTCCCGGgTCAGTCCCTCTA

AGAGGGCCCcAAATCCCCGAGGTACCCTCAGGTGAGTGTTGGAAAAaGGCACTGACATTT

TCCTGAACCACGTGTCTGATTCCAAGCTATAATAACAGCAAAAAAGGTTTCCGGCTTTTC

ACAAGTGAAATGTCACCACTAATTTAGCATCTGTAGTTAACGGACTTTCTAAATGTTGTT

TGGGTTCGTAATTAAATCGCGCTGTCGAATCGGTTAACTGTTTAAATGTAAACCATCTTT

CTTAAAAATCCGTTCTAATACAACGGTttGTTGTGACCTGGTAGCATTTTAAATCGACCT

TTACCGAATCGTTTATAGTTAT

>contig30993

GTCtGCCGGACCCGAGTCCTTCCGCATGTGGGCCGACCTGCGAAACTtCCTGCTGCAGCT

GTGTGAGAACCTGACCAAGTCGGCTGAGGCCAACTCTCCGGCTCATGAAGATTTTGAGCA

GATGCTGCTGATCGCTCACTACTTGGCCGCGCGATCCGCCGCTAAAGGAGTCGAGCAGCT

GATGAGCATAGCAGCGAAGGTTTCTGTGTCTTTGCTGCGTTACACTGAGCTGATTCGTGC

AGATAAAGCTTTCTATGAAGCTGGCCTAGCCTGCAAGGCTGTTGGCTGGGAGAACATGGC

ATTTATCTTTTCAACCATTTtGTGGACTTGTGTGATGCGTTGGACGACGGCCGCGCTCCG

GATCACTCCGACTTCCTGGACACAGACATTCCCTTCGAGGTCCCGCTTCCCACCAAACTC

TGCGTGACCGACGCCCAGCTGGAGCAGATCCGTGACTGGGTTCTGATGGTGTCTGTGGAA

AACCGGCTGGAACAGGTTCTGCCTCGGGACGAAAGGGACACGTACGAAGCGTCGCTGGTG

GCCGCCAACACCGGCCTTCGCTCGCTGCCGTGTGTCCTCACCGGTTATCCTGTGCTGAGG

AATAAAATTGAGTTTCCTGTTGCTGGAAAAGCAGCCAACAAAGAAGATTGGAATAAATTT

CTAATGGCAACAAAGACGACACACAGTCCAGAGTGTCAGGATGTCCTAAAATTCCTAAAT

CACTGGTGCGGTGGTCTACCTGCAACAGGATTCTCTTTCCACTAATGGGACTCCGTACAT

ACTTCCATATTCCTTAGCTGCTACTCTTTCTCTTTGTTTTTTCGGTTGTTTCTCTGCCAG

CTTTGTCAGaTTTTTTtAAAAAATTtaTTtATTCTGTTTTATTGAAGTTAAATGTTGTTT

AACTGTACTGTTTACGaGAaCATAAACATTTTAAATTAAACTGAAGAAATAA

>contig32045

ttcAACaTAAGCAAACaTGGCTTCtGACTACATTTtCCAGTTTtGTTGTCTCTGCAGTGG

ATCGCTCCCCAGTGACAAATGTTATACCTGAAATCGCTCCATTTAACATCACTGAACACC

GCTCTCTATAAAGTTCCCTCACCAAAAGTTAGAAAATAGTATCAAATATTAAAACACTAT

TAAATATTAAAAAGTCTTTTGTACTATTATTATTCTAAATTTTACATAAGGTATAAATAT

GTGAAGAGGATGGGGATATTTATTTCTGTAATAAGAATGTGCAAATTGGCAGCAGCAGAA

ATGAATAAACTATCACAAAAACTCCACACCAACTCAGAAGACTTGAATGTGTAAATAGCA

GGCAAAGACTGAAACATATTAAATATTATCAGTGCTAATATCAAGAAGGTTCAAAATTGG

TTATTATCTGAGAAATAAAGTATAAATTAAGACAACCGACTGGAAAACAGGACCCATGTT

TCAAACAATTATTTTGCCATATTCATTTTGAAATCCTATCTATGTTTTGTGTACATAAAA

CCATTATGAAAATGGTTGGAGTGTTATGAATAAAGCATGTACATATGAGATGTTTACCTA

CAGAGACAATACCTATTCATATGTATGAACCTGAACGCAAACATTAAAAGACAAACGTCG

TTGGGGTGTGTGGGGATAGCaGAAGCCGAGAGCGTCAAACATATTTTCATAGCTGTTATT

ATCGTCATTAGTCAGTTAGAGGATTCATTTTCTAAATCTGTCACCACCAAATGAATTCTG

ATGACAGTGATTGGTGCACTAATCAGTAGCCCCAGTGAGCTTCTTCCTCGCAACACATTT

TAATATCCCCCTCCCCAAaGCcTCCcATTtCTTCTTTGAtGTTTAAACCaGCAAAA

>contig33067

tttCCAGGGATTGCACCTGCATTGCTAAGGGGGATGCTCGTTTCACGCTCTTATGCGGCA

TCAGAGACTTTCAGTTGTTGCGAAGATAgactgaggatcaacaaaagataactcgcttca

tgctgctcattgggatccacgCCAATATGGATGGAACGGTCCTTTATGAGATGGCTGCTG

CCCTGTTCATTGCTCAACTCTCAGGCTCCAGGttgcactggagcaagatcatcagcattg

ctgtgACGGTGGCAGTGGCcACTCTTGGAGAGGCAGGAATCCCCGCTACAGGAATGATGA

CCACCCTCTTtATTTTGACCATCAGTGGGATTCCAGTGAGACCTGCCTCCCTTTTGCTCT

CCATTGAATGGCTGCTaGATCGCCTCAATGCCGCTGTCAATATCATGAGCGACTGTTTTG

GTGTCTTTCTCGTtGCTAATGTGTCAGAAGGAGAACTGAAAAAAaTGGAAGAGGAGATGC

CTTATGAGCATCTACCAGAACCGTGGAAGCCACCAGAGGAGCTTGCTTAAACAGCAGCCC

TTAGCAGTCCTCAACAACGACACGATTGTTGACAGTTGTAAAGATGAAGCGATGGACTTC

AGCATTtCtATTTTTtGTGCCATTTGCATATTTATCACCTTTGTAAGTTtGCCTTGTAAT

GGTATTGCTGTTATTTTAAATGAGCATGCTGACAGTTATTTCAATATTGGGTTTATTTGA

GGGAAAATATATATATTTAAGTTTGTTTTAGTTTTTTTTtAGTTACTTTTATtGTTTTTG

TCATTTGCACTTAtAGCATTTTACAGTACTACGATACTTTTGGATAACTTCAGCATTCTG

TCATTATATATGATGTTGTTTGGTACTAAaGAAACAATAAACGTGCACTTGAGCAGaaaa

aa

>contig35727

TACCTTCACTCGGCGGCATAGCCACTTtCCTTTtCtGATCAGCTCAGAAATGCTGGCTGT

TTAAGGGACGAGGCTCCATGGCACAACGTCTACGGTGAACgCTGGCCTGTTGAAGCGGCA

GATGGAGGATCCAAGTGGGACCACTGACATTCACTACGAGGAGGGAGTGATTGTTTTATC

ACCCTACAGAGGTCCCATCAAGTGTGAGGATGTCCTGCAGCTTCTGCACAAGACCGCCAC

GCCGACTCACATGGGCACCCTTCCACGGGGgTCGGCTCCTTACATTACACTGAATCACGC

CACAGGAGTCACGGGGGGCAACTTGACCGCCGTGAGCCAACCAGCTGTATGTCAGCCCGC

CCCcGCCCTTCAATCTTCATCCCACTACTGCGGCAACTGTGGATGTAAATCTGAGCATCC

TTTAGGTTGCTGCTGTGGGGCGCAACTTGTCCCTCCTCACATCTGTCAGAGCGGCGGCCT

GCCTCACCCCATCAAGAGGCCTCCGCTGACCGAGTATCAGGACAGCTATTCCGCTAAGTG

GTTCCAACCGAAGATTAAACCCGGCTAACGTCACCTTCCTCCTTGTCaCAAAGTTCAGCC

CCCAATACACAGCTCATGAAAAGaa

>contig39984

AAACTTCTCATCAGCAGTGAAGCCCAATATGTCAATAGCAGTATCTGTAGCGATGAACTC

CTCCACATCATCAATACTCTTGACAGTGATTTCACCCTGGCTGATCATTGGGAAGTCATA

GGGGTTGGTGGTGATCAGCAGAGCATCAATGAGCTCAGGCTTGTGGGCTGTCATAAGCTG

ATAGAAAATATGGTAGCTCCTCTCAGCAGACAGCTGGAAGGTGACACGGGACTTCTCCAG

cAGATATGTTTCAATATCAGCTGAAGCCAGCTTTCCACTTGTACCAAAGTGGATCCTGAT

GAATTTACCAAAACGGGAAGAGTTGTCATTCCTCACAGTCTTGGCATTACCATAAGCCTC

TAGCAGAGGGTTAGCAGCAACAATTTGGTCcTCAAGGGAGCCCTGGATTTTTCCCGCACT

TTGCGGTGCTTGGTCCTTTTtGCCACCAAGAGCTGCAATTGTTGCAAAGTACTGGATGAC

ACGCTTGGTGTTGACAGTCTTGCCAGCACCGGATTCTCCGGTAATCAGGACAGACTGGTT

CTCACGATCTGTGAGCATGTACTGATAGGCATTGTCAGAGATGGAGAAGATGTGAGGTGG

AGCCTCAATCCTCTTCTTGCCTCTGTATCcTGCTACAACAACAGCATCATAcACAGGCAG

CCACTTGTAGGGATTCACGACCACACAGAACAACCCGGAGTAGGTGTAGATCATCCAGGA

TGCATAACGCTCTTTGAGGTTATACAACACACAAGGCTCGTTGAGGTGGgTCATCATGAC

CATGTCCTCAATTTTGTCgAaCTTTGGAGGATtCcTGGgaTgAaTaTCATCCTCTTTGAC

TGTTACAGTCTTgCCTCcTTcTGtTCAACAGTGGCTTTGCCACCTTCTTTCTTCACAAGT

TTCCCCTTGAGATACATCTCATCAGGATCcACCACAAaGAAAGCTGaTTTaGCATCAAAT

GGAGCGGCCTg

>contig40011

ttAAaTAAACTAAGGTCCTTTCGTACTACTAATTCTTTTAAAACTTATAGATAGAAACTG

ACCTGGCTCACGCCGGTCTGAACTCAAATCATGTAAAATTTAATAAGTCGAACAGACTTA

TTTCTATAATTTCTACTTCATAGAAAAATTTTAATTCAACATCGAGGTCGCAAACATTAT

TATCAATATGAACTATCCAATAATATTACACTGTTATCCCTAGGGTAACTTAATCTTTCA

TCCTTAAATAGGATCAAAATAAACTATAAAATAATTTGTTAAAAAATTAATCGTCCCAAT

CAAAATTTATACAATCAATTAATTATATATTATGAAAAATTTATTAAAATTAAAGATCCA

TAGGGTCTTCTCGTCCCATAAGAGCGATAAAAAaTTTTAATTTTAATTTTAAATTCAACT

TTATTCACAAATCATTAATATTTTtCCATAAAACCTTTCATACTAGTTTTTAATTAAAAA

aCAAATTATTATGCTACCTTAGCACAGTcATAATACTGCGGCCATTCAAAATTCATCGGG

CAGGCTTTATCTTTTATTCTCCAAAAGAAAATGTTTTTGTTAAACACTTAAAAAAaTAAT

TGTTAAATTCAAATTATTAATATTTTCGTTATTTATTTCTACAATTTATTTACTAATTAA

AACATAATGACCAAATATTATAAAATAAAATTAGTCAAATTTCATAAATAAACTAAAAAA

AATCATAAAATAATCTAATTAAATTTTTAAAAACTTTTAATGCCTATTATAAAGACAATA

AAATTAAATTAAATTAATTAGCATAAAAAAATTTAAAGCTCGTCCCTTAAATTATTCATT

TAATTAAAAATAATTTTAAAAATTATAAGAAATTAAAAAGATATAAAAGAATACGAAAAT

TTTtCATTTCCACCAACATATTAAATAAaTTATAAAaCATAATTTTtCATTTGCTGGTAA

ATCATTCTTTTTCGTTTAATTAAATTTTAAATTTATTATCTCATCCTGATAc

>contig40031

ttttttttCACATTAAATATTTTTATTTATTGTAACATTTGATCAAACAATCTGACAAAT

GGATTTAAACACGTTTCTTCTCTGCCTCTCATATCTGTGTTTTAGAATACAGATTTTCAT

AATTTTTTTTtAAATGCAATGCTTTTATGCTTTGTTATTATCTTGACACCTCTTAAGAAA

AACACCAACACCTCTGTGTTTTTGACACACAAAATACATCATTTCTAGACAAATAGTCAT

CAGATGTTTTTGACACAGATAAATGGCCTTCGAAAGTTACACTGCGCATCGTCCCAACAT

CTGTGCCGACTGTAGTTTATCTGCAAACAGTCcTGATTAGCTAAGTTGTTGGGTTCGTAT

CGACACCAATTACTGTAGTTAAAAGGGCTTCCGTCCACCCAGCGCCAGTTGCGCTCCCAT

ATTATGTCATTGCCTCcAATCCAGACTGCAGTATGCCCCCGGCCTGCAGACATTATCATC

CTCACAAGCTGATTATTCACCTCGTAGTCATGGATTGATGCAAGGTGTGCTTGTATGGAC

CTGCAGTTCTCCTGAGCTCTATGCCAAGTAGCACCAAAAGGAAAGaCGTGGTaGCAGCGA

CCTTTGACCCGGGTCCAGCCAGAAGAACAGGACCCTGAACGTCGGACTGTTCCATTCAGA

GCccTTCAGGTAAGCTACTTCATCCACAGGTTCAAaTGTTGGTTCTTCTGCACCTGTCCA

GTTTCCAGGTTCCTCTTCATGAAGAGCCAAATGGTCTTCTGGAAAAaCGTCCTCAGTGTC

AGGATCTTCTCTGGTTCCTTCTGGAAGAGCTTCATCGCTCTCAGCCTCATCcATGGTATC

TGGAAGCGCAGCAGGTTGACTCAAAGCCAaCAAGGCACAAAGAAGCAGCAGCAGACTGAG

AGCCTTCATGGCTTTGATGATGCAGGTTGAAGACAGTAAAGAGATAATAAGGAGATAATA

GAAGCTAATTtCGAccAATCACACCACAGGATATCCTCCAGGTTACAGTCAg

>contig40414

TTTttGAAaGTGTAGTCTGTTTTAATAAGAAAGTTTTTGCAGTGTACTTTAAAAATCGTG

ACATTCACAGGTTTTAAAGGTGCTTCAGACAAGAAATGAAATGAAAAATAAAAGCAATTT

CTCACTAAGAAATGTAATTGAACAGAGGGTTCTGTTTTCCGCTATGACACACACCACTGC

TTCGAGTTAAAGtAGCTGAGTCGTACAACACTACAATTCATAAGAATCCTTTCCAAAGTC

TTCATATCAAATTACAGTCCTTAAAAACATGCATACCTTTTGATGTGAAATTTTAAATGG

TTCAAAACAAACATACTACAGTTGAAATTATTCTAGTTACAGCAAAAAGCACAACTGCAC

AATCACAAAAGAAACTAAAGAGAAAAGATTTATATGAAAACAACTTCTtCTtTTTTTTTT

tACACAGGAAGTAATCATCAAAGTGGAGCCTGAAGTCGCACTGTGAACTCATTGTGGATA

CTTCTTGTCGCAAGCTGAATCAATCAGTGAGCTGAGATCTAAGAAGCAAGTCCCTGTGCT

GTCCTGCTCATCTTAATCCTGAACCGAGTTGCTAACCTCGACGTTTACGAGCTCGGCATC

ATTGTCTTGATCAAACTGAACCTCATATATCACATCTTCAGCCATTTGCTCAGGTACTGA

AACCaCAAGCTCCTCATTCTCCTTTTTAACTACAGGAGCTCTGCTGTTTTTAtATGCGAG

TGCATCCCTGGGGTCAGAGGAAGGCTGTGGCACAGAGACGGGTTTCTCTGCGCTCTCCAC

TTTCACCAGACAGGCACTGGGTGAaGTTTGCATCACCTCATCTGCAATCTGGAAAACTAC

ATccAAGTTAACTTCAGGCTTTTTCACTTCCTGGCTTTCGGAAGTTTGTAaGTTGCGGTT

GGGGTCGAACACGGTCATG

>contig45477

acgACAGGAAACCTTTGTGGTTCAACCcAGCTTTCTTTCCCTACCCCGCCCCGATGAgTG

AGCCCcTCGAGCACAAGAAACCTGAATTCGATGACCTCCAGGATTCCAAGTGATATGCTC

TTTCGAGTCAAtCAGAGCCGACGAGTGTT

>contig41732

TAATCTGCGTAACGACGTGGTATTCCAGCGAGACCTAAGAAATGTTGAGGGAAAAAGTTA

AGTTTACTCCTAAAAATATAATAAAGAAATGTACCTTAAGAAGAAACTGATTCATTCTTA

ATCCGGTCATTAGAGGAAATCAATGtACAAATCCTGCTATGATAGCAAAGACAGCACCCA

TTGATAAAACATAGTGGAAATGGGCAACTACATAATAAGTATCATGGAGAACAATATCAA

TTCTAGAGTTCGACAGGACAACTCCTGTAAGCCcTCCcACAGTAAACAAAAATACAAATC

CTAGGGCCCACAACATAGATGGAGTTATAGTAAGTCGTGTCCcGTGTAAAGTTCCGATTC

ATCTAAAAATCTTAATCCCCGTAGGAATTGCAATAATCATAGTTGCAGCAGTAAAATAAG

CTCGAGTGTCCACGTCCATTCCcACAGTAAaTATATGATGAGCCCAGACAACAAAaCCAA

GAATACCAATTGCAAGTATAGCATAAAtcATACCTAATGTACCAAATGCTTCCTTCTTAC

CTCTTTCTTGGCTAATAATGTGGGACACTATCCCGAATCCAGGTAAAATTAAAATATAcA

CTTCAGGATGGCCAAAAAaTCAAAATAAATGTTGATAAAGGATGGGATCCCCACCACCTG

CGGGgTCGAAGAAaGAAGTATTTAAGTTACgaTCAGTTAACAGTATAGTAATAGCCCCCG

CTAGGACTGGAAGTGATAAAAGGAGAAGAACGGCGGTGATTCCTACTGCTCAAACGAAGA

GAGGTATACGGTCAATAGATATTGACTGGGgTCGTATATTAATGATAGTAGTAATAAAAT

TTACAGCCCCTAAGATAGAGGAAACTCCAGCTAAATGAaGCGAGAAAATAGCTAAATCTA

CAGAAGGTCCGGCATGGGCAATGGCTGAGGATAGAGGGGGATAAACTGTTCATCCAGTTC

CTGCACCTCTCTCAACTaTAGATCTGGCCAAGAGAAGAGTCAAGGATGGTGGAAGTATTC

AAAATCTTAAATTATTTAACCGGGGAAATGCTATATCCGGGGCCCctAATATAATGGGTA

CTAGCCAGTTaCCAAATCCCCcAATCAAGATTGGTATAACCATGAAAAAAaTTATAATAA

ATGCATGAGCTGTCACAATAACATTATATACTTGTTCATCGCCAATCAGGGAACCTGGTT

GACCCAACTCTGCTCGAATGAGCATTCTTAAAGAAGTCCCAACTAt

>contig43161

ACAGACCTGCAGAAGCAGTTGGTACAAtCGGGAATTTGCTTTGAGCCGGGCATTCACCAT

CATCTTCATCTTGCTCGCCTCTTTCATCTTCCTCAACATGTTCGTGGGTGTGATGATCAT

GCACACAGAGGACTCCATCAGAaaGTTTGAGCGAGAGCTGATGTTGGAGCAGCAGGAGAT

GCTCATGGGAGAGAAGCAGGTGATTCtGCAGCGGCAGCAGGAGGAGATCAGCAGGCTGAT

GCACATACaGAAAaaTGCTGACTGCACAAGTTTCAGTGAGCTGGTGGAnnAACTTTAAGA

AGACCTTGAGCCACACt

>contig43726

CGGGTttGtCTGTaGGATCTATACTGAAGTACCGCCTCAAGTTTTCTGaCTAATTCAACT

ATGTTAAGATATGAAATTCCTGTGTTTTGGTTGTTTCCTCAAGATGTTTTCATTGTTTAC

AATGAGGATCACTATTGAGGAACTCCAAAGCGATATAAACAATTTATTTATTGTTCAACT

CATGTTTATCTCAACGTTTTTCATTAATCAAATTTGTTTTTTTTt

>contig43740

CTGATAAGAAGACTtGCAGCGCTcGTAGACCGGCCGACCAGCTAaAGAACACTGAATGCT

TCACATACGCAGAGGAAGTTGGTGAGAGATGCAACGGGAAACAGTGGTGTAAAGTCAAAG

CCAGTAACTATGTGTTCGGAGATCCCTGCTATGGAACCTACAAGTACCTGGAGGTGGAAT

ATATTTGTTACGAGGTTCCCCAGTCGTGCCCAGAATAGAAGCAGAAGTCGCCTTCTTCAT

CAGGATGcTGATGAAGACTCTGGTGCTGCTGCCCCAACAGATGATGGCTGAAGACAACAA

ACTGAGAGAAGATCTGCAGCATCTTGTTGAAAACCTGGAAGGATCTTAGCGTCCTCAAGA

AGCTGACAGAAAATAATCTGAATTACAATTAAACATAACAAATAAAGTAATATCACAGAT

TATAGCTTTGAAAATCTTTTTATTTTtCTTCCTGTTGTATCAAACTTAAAGACGgTTTTT

CTCTCCAACTGCTTCTCTTTGTAAACTTCTTCAGCCAAAaTAAAaaCAAAAAGCTGAAAG

AgC

>contig43741

GCGGGGCACACTgCTGCTAGTGAATCTGGTTTCTGCTGCTGCTTTgATTCTTCATCACAA

cAaGATGAAATTTGTTCTTTTTACTTTGTTCCTTTGTGGATTCATCCTGGAAATCAATGG

AAAAAGTTCATCAGGAGCATGGCTCCCAGaGAAAAAATCCCTTGTTGCCTGTGAAGGAAC

AGTGGCTCGACTCCACTGTGAAGAAGGTCAGGCTATCTATGTGACCAGTGCTACATACGG

ACGCA

>contig44129

aacgcttggtgaattctgcttcacaatgataggaagagccGACATCGAAGGATCAAAAAG

CGACGTCGCTATGAACGCTTGGCCGCCACAAGCCAGTTATCCCTGTGGTAaCTTTTCTGA

CACCTCCTGCTTAAAACCCAAAAAGTCAGAAGGATCGTGAGGCCCCGCTTTCACGGTCTG

TATTCATACTGAAAATCAAGATCAAGCGAGCTTTTGCCCTTCTGCTCCACGGGAGGTTTC

TGTCCTCCCTGAGCTCGCCTTAGGACACCTGCGTTACCGTTTGACAGGTGTACCGCCCCA

GTCAAACTCCCCACCTGCCACTGTCCCCGGAGCGGGTCACG

>contig44670

aCCGaCTTTTTGTtAGATGTCCACATGAACAGCTTTGCTGTAACGCAGGCTCAGTCTGTC

CAGCTTTGGGCTCAATAGTTTCATGGCAACGTTGCAAGCAGCGGCAACATCAGGATCAAT

GGTTGCATGGTTCCTACTCAGGGACTTCATGTGAGAGTAAGTGAAGCTGGCCACCTGCAA

ATTCTCCTCTGTTTTCACAGAGTTGGCAACAGTGGCCACCAAACCCATGGAAGGACTTGT

CTCGAAGAGAACAATGCAGGAAAGCATACGGAGCTTTGGGTGGAGAACCTTGTCCATGTA

GAGCTCGAGAACCAGATCCTGGACCAGTCTGGGCTCCTTCTTTGCAATGTTCCTCAAAGC

CATGATGGCTTCAACATGAATTCTCATTGGCAGAGATGCAGCGGCAGTGCCATGGATGGG

CATGATCTTAGTGAGAGACTTGAAGCTAGATGGATGTCCAGCATTTCCCAAAACCTTTAT

GTACAGGATGATGTTTTCTTCCTCATTCTTGGCAACAGCATTTGAAAGTCGTTCTTGAAT

AGGCTTTATAAGTTCAGCTGGACAGGCTGCTGTCTCATTACAATATTTGTAAACCATTGT

ACCGTATCCAAGGAAGACAATCTCACGCAGAAGTGGATTTCTCTCTACTTTGTCGCTCGC

TAACAGGTTCTCGAACAGCTTGATAACCTCGGGGTCAGCAGTCACCATGTGCACAGCTGC

AACCAATGCTTGAACAGCCTCAGCAGTGTTTATTTCCTCAGCCATGAACTTCTCTTTGAT

GAATCTAAAAGCAGCGGGAGTGCCAGCAGCAGGGATGGTATCCAAGAACCAGTGTCTGTG

aGGGGACATCTTTTTGTACTGGTTCCAGTACATTTCCAAATCTTCGTACCCGAC

>contig45151

CTGTCATGGAATCTGTCAAGCAGGGGGCTCGTAACGTTGCCGACAGCGCCAAGAACCTCG

GGgAGAAACGCCAGTGAAGAGGAGACGACAGTGAAAGGCGAGACGACGATGGAAGATGAA

ATCTTGGATGAAATCGAAGCTCTATGCTTTAGTTCTTGA

>contig45247

ATCAGTCATCCTGCACTGGATCTGCTTTAAGCCGCAGCCTCTGTGGCTGGTGAGTTTCTC

TTGACGCAGACAGACGGCAGCTTAAGAGAACACTTCACGTCATCCCAGCaCGTTTTTTTG

AATAGTTCATCTGTATGCAGTTCTGAAAGCCATGTCCATTAGGCTGTCCTGGACACCAGT

TTGTGTAGGTCATAGGGGTTCCATCGCTCCACAACCAAATGTTCTCCTCCTGTGCATCAG

TGCCTCcGATCCATGTTATACTGGGCTTATGACTTGCCGTAAATATCAGAGTCTGAATCT

GAAGGTAATCCTCTTT

>contig45262

GTAGCACATATTGATTTTtATAAAAGCATAAACAAAAAAAGAAAATGAAaGAAAATACTC

AATAAAATAGAGGTAGACAGATTCGAGTAACTCATCGGTAGCTATTTTTTTTtCTATtCT

ATCAGCATTCTTTCGTGTTTTCTATTGATGAAAACTTGAAAACAAATTTTTTAACCTTCA

CTAACAGCACTGAGCAAAATATTTTtGAGGATTATCGTCAAGTGATAAA

>contig45318

ttttATTCTCTTGCATAGAGAGTCATTAGAGTCATAAAAACATAAGATTGAATAATAGCT

ACAGAGAATTCCAACATTAATAGGATAATCTGTGAAAaTACTACAATTGAAGTAACATAT

AAATTTTCCAGGGTACCTTGAGCCCCTAAAAGAGTCAATAGGAGATGGCCGGCAATTATA

TTAGCCGCAAGACGGACAGAGAGTGTAATAGGCCGAATCATATTTCTAATGATTTCAATT

AATACCATGAAAGGTATTAAAGGTGCCGGTGTTCCTAAAGGTACAAGATGGGCTAAAGCA

TTCGTTGTCTCCTTAATCCAAGTATATAGAATAAAAGAGATTCACAGAGGAACTGCTAGT

CTTAAGGTAACTGCTAAATGTCTAGTAGCGGTAAAAATATAAGGGAATAAACCGATAAAA

TTATTAAATAAAATAAATAAGAAAAGGGCAATAACTAGGATATTGGCACCAAATCTTGCT

GGGCCTATAAGGAGGGATATCTCCCGATTTAATCCCATTAAAACTCTCTTAGCCAAAAAT

TGAGGTCGCGAAGGAATCAATCaGAAAGATATTACTATAAACAAAAGTGGGATTAGTATT

CTTAATCAG

>contig45522

AAAAGGGATTCGGTGCTCATTTTTTCGTTTCTCCCATTCCCTTCAAAGCACCTTTCAATT

CACTATTTCAAGTTCAATTCTGCTGAATAAAATTTGACAAATTGCTTGAAGGGATGGCGA

AGACAAATCGTGAGTGATCCCTTTGAGTTGGTGTGGATGGATTACGCTTTCTATTTTAGA

TCCTGTCAGAGTTTTTGAATTCAACTAGTAATGTTTTTTCGTGGATTTATTGTTCTGTTT

TTAATGTCGAATGGACGAGGAATTTTGATTAGTAATTCGA

>contig45523

AAAGGGATTCGGTGCTCATTTTTTtCGTTtCTCCCATTCCATCAAGCACATTTCAATTCA

CTATTTCAAGTTCAATTCTGCTGAATAAAATTTGACAAATTGCTTGAGGGGATGGCGAAG

ACAAATCGTGAGTGATCCCTTTGAGTTGGTGTGTGGATGGATTACGCTTTCTTTTTTAGA

TCCTGTTAGAGTTTTTGAATTAAACTAGTAATGTTTTTTTCGTGGATTTATTGTTCTGTT

TTTAATGTCGAATGGACGAGGAATTTTGATTAGTAATTCAA
